# Unguided bibliotherapy and digital self-help interventions based on cognitive behavioural therapy for university students with gaze-related anxiety and social anxiety symptoms: a randomised controlled trial

**DOI:** 10.1016/j.invent.2026.101023

**Published:** 2026-11-06

**Authors:** Kazuki Matsumoto, Sayo Hamatani, Yoshihiko Kunisato, Katsunori Watanabe, Shinobu Nagata, Akiko Maeno, Mizue Yokoo, Yasuhiro Kimura, Naomi Sunami, Masatoshi Ikeda, Makiko Kasai, Rio Kamashita, Yoshifumi Mizuno, Masayuki Nakamura

**Affiliations:** a Division of Clinical Psychology, Kagoshima University Hospital, Kagoshima-City, Kagoshima, Japan; b Research Center for Child Mental Development, University of Fukui, Yoshida-Gun, Fukui, Japan; c Department of Psychology, Senshu University, Kawasaki-City, Kanagawa, Japan; d Department of Psychology, Jin-ai University, Echizen-City, Fukui, Japan; e Clinical Psychology Course, Department of Psychology, Shujitu University, Okayama-City, Okayama, Japan; f Department of Clinical Psychology, Faculty of Human Relations, Shigakukan University, Kagoshima-City, Kagoshima, Japan; g Psychological Course, Faculty of Modern Communication, Tokyo Rissho Junior College, Suginiami-Ku, Tokyo, Japan; h Department of Welfare Psychology, Faculty of Welfare, Fukushima Gakuin University, Fukushima-City, Fukushima, Japan; i Department of Psychology, Faculty of Liberal Arts, Teikyo University, Tokyo, Japan; j Professional School of Teacher Education, Niigata University, Niigata, Japan; k Clinical Psychology Course, Naruto University of Education, Naruto-City, Tokushima, Japan; l Division of Occupational Therapy, Department of Rehabilitation, Hiroshima Cosmopolitan University, Hiroshima-City, Hiroshima, Japan; m Department of Psychiatry, Graduate School of Medical and Dental Sciences, Kagoshima University, Kagoshima-City, Kagoshima, Japan

**Keywords:** Social anxiety, Cognitive behavioural therapy, Randomised controlled trial, Bibliotherapy, Internet-delivered intervention, Treatment adherence

## Abstract

Individual cognitive behavioural therapy (CBT) for social anxiety disorder is effective, but CBT has not become widespread enough. Since young people with social anxiety also experience above a certain level distress and functional impairment, the development of readily available CBT-based self-help interventions is urgently needed. The objective was to evaluate the effectiveness of two five-week, unguided CBT-based self-help interventions in reducing gaze-related anxiety and social anxiety symptoms among university students with elevated gaze-related anxiety. A three-arm, randomised controlled trial was conducted in Japan from April to December 2025. University students with gaze-related anxiety were randomly assigned to bibliotherapy (Bib) or unguided digital self-help interventions (DSI), or to a six-month waitlist control. Social anxiety symptoms were evaluated using several self-report scales, and assessments were conducted at baseline, post-intervention, and at two follow-ups. Of 252 university students enrolled, 244 were included in the modified intention-to-treat analyses, which used linear mixed-effects models. For the primary outcome, the adjusted mean difference in the Gaze Anxiety Rating Scale at post-intervention was −10.09 (95% CI: −14.61 to −5.56, Hedges' *g* = −0.66) for Bib and − 6.95 (95% CI: −12.11 to −1.79, Hedges' *g* = −0.47) for DSI, both showing a significantly greater reduction compared to the waitlist control. Similarly, for a secondary outcome, the adjusted mean difference in the Liebowitz Social Anxiety Scale at post-intervention was −13.57 (95% CI: −20.86 to −6.29, Hedges' *g* = −0.55) for Bib and − 12.47 (95% CI: −20.78 to −4.17, Hedges' *g* = −0.53) for DSI, which were also significantly higher than the waitlist control. The effectiveness of these two interventions in reducing social anxiety symptoms compared to the waitlist control was maintained at the 3-month and 6-month follow-ups. These findings support brief unguided CBT-based self-help as a potentially accessible low-intensity option for university students with elevated social anxiety symptoms.

## Highlights

• Five-week unguided self-help reduced gaze-related anxiety in university students.
• Bibliotherapy and digital self-help reduced social anxiety versus waitlist control.
• Between-group differences remained evident at 3- and 6-month follow-ups.
• At 3 months, response was 41% with bibliotherapy, 36% digital, and 22% waitlist.

## 1 Introduction

### 1.1 Background

Social anxiety disorder (SAD) is a common psychiatric disorder that typically begins in adolescence and often follows a chronic course if untreated (Stein and Stein, 2008; Solmi et al., 2022). Social anxiety can be conceptualised as a spectrum ranging from subthreshold symptoms to clinically significant SAD (Schneier et al., 2002). Young people with elevated social anxiety symptoms may experience substantial academic and interpersonal difficulties even without meeting diagnostic criteria for SAD (Jystad et al., 2021, Jystad et al., 2023). Given that SAD frequently develops during adolescence, accessible early interventions for young people with subthreshold symptoms are particularly important (Chronis-Tuscano et al., 2018; Zhong et al., 2024). Individual cognitive behavioural therapy (CBT) is recommended as a first-line treatment for SAD (Mayo-Wilson et al., 2014; National Institute for Health and Care Excellence, NICE, 2013). However, limited therapeutic resources make it difficult to provide individual CBT to the broader population of young people experiencing elevated social anxiety symptoms (Jefferies and Ungar, 2020). Low-intensity self-help interventions may therefore offer a scalable approach to addressing these unmet needs.

### 1.2 CBT-based self-help interventions

CBT-based self-help interventions include bibliotherapy (Bib), which uses structured self-help books containing psychoeducation and CBT exercises, and digital self-help interventions (DSIs), which deliver therapeutic content through digital platforms. Both formats can be delivered with or without therapist guidance. In the present study, Bib refers specifically to a printed CBT self-help book, while DSI refers to a web-based or a mobile-application CBT programme.

Previous randomised controlled trials have suggested efficacy of printed self-help books for social anxiety. In South Korea, Chung and Kwon (2008) recruited participants through a social phobia counselling centre and assessed eligibility using semi-structured interviews conducted by clinical psychologists. Thirty-eight participants were randomly assigned to a six-week printed CBT self-help book intervention with therapist feedback on homework assignments via email (*n* = 13), the same intervention without therapist feedback (*n* = 11), or a waitlist control (*n* = 14). Both Bib conditions significantly reduced symptoms measured by the Social Phobia Scale compared with the waitlist control. Similarly, Furmark et al. (2009) conducted two randomised trials in Sweden, recruiting participants through media advertisements and internet-based sources. Following online screening and structured diagnostic interviews by telephone, participants were assigned to nine-week self-help interventions or comparison conditions. The Bib significantly reduced social anxiety compared with a waitlist control. Krafft et al. (2020) also reported improvements in social anxiety among university students who used traditional CBT or acceptance and commitment therapy self-help books over eight weeks.

Unguided DSIs have been suggested to be effective in reducing social anxiety symptoms (Mayo-Wilson et al., 2014). In a previous study, 200 university students in German-speaking countries enrolled in a clinical trial. Participants who met the diagnostic criteria for SAD were given the opportunity to complete all nine sessions of the DSI independently. In this trial, the DSI significantly reduced social anxiety symptoms compared with a waitlist control group (Kählke et al., 2019). In Japan, a previous study involving 77 high school and university students showed that a DSI significantly reduced social anxiety symptoms compared with the no-treatment group (Matsumoto et al., 2024).

### 1.3 Brief psychological interventions and remaining evidence gaps

According to more recent evidence, psychological interventions such as CBT may be effective even when the intervention delivered over shorter periods. An umbrella review suggested that single-session interventions could reduce psychological symptoms; however, only a few randomised controlled trials have targeted anxiety (Schleider et al., 2025). A systematic review and meta-analysis suggested that brief group CBT, including single-day interventions, may reduce anxiety symptoms (Zhou et al., 2025). However, the included trials in this review varied considerably in intervention content, target populations, treatment settings, and outcomes. Furthermore, most of the existing evidence was generated by therapist-delivered or group-based interventions, thereby limiting its generalizability to the effectiveness of unguided self-help.

Previous studies evaluating unguided self-help interventions for social anxiety have used treatment programs lasting 6 to 10 weeks (Chung and Kwon, 2008; Furmark et al., 2009; Kählke et al., 2019; Matsumoto et al., 2024). Self-help intervention programs designed by CBT experts often include intentionally edited features to support independent practice. These features include clear and simple language, illustrations, diagrams, instructional videos, step-by-step goal decomposition, illustrative case stories, self-monitoring tools, techniques for cognitive and behavioural restructuring, detailed implementation guides, and downloadable module materials and worksheets (Redding et al., 2008; McCall et al., 2021; Hedman-Lagerlöf et al., 2023). These elements help users understand therapeutic concepts and maintain motivation without continuous professional guidance.

Unlike therapist-led CBT, self-help interventions allow individuals to work through materials at their own pace and revisit specific exercises as needed. This flexibility encourages repeated practice of CBT skills and enables users to target difficulties most relevant to their symptoms. However, to our knowledge, no study to date has evaluated the efficacy of unguided self-help lasting 5 weeks or fewer. Given that early intervention and prompt symptom reduction remain a clinical priority for individuals at risk of anxiety disorders (Moreno-Peral et al., 2017), developing brief self-help interventions capable of rapidly reducing social anxiety symptoms is highly valuable.

Another critical gap in the literature concerns gaze-related anxiety—a phenomenon marked by distress, discomfort, or avoidance during eye contact or when feeling observed. Although not classified as a distinct psychiatric disorder, gaze-related anxiety is prevalent among individuals with SAD as well as nonclinical populations (Chen and Clarke, 2017; Schneier et al., 2011; Alencar et al., 2023). Given its close links to self-focused attention, avoidance, and fear of negative evaluation (Harbort et al., 2013), gaze-related anxiety represents a promising target for early CBT interventions.

### 1.4 Objective of the present study

The present study aimed to evaluate the efficacy of two five-week, unguided CBT-based self-help interventions—printed bibliotherapy and a digital self-help intervention—in reducing gaze-related anxiety and broader social anxiety symptoms among university students with elevated gaze-related anxiety, compared with a waitlist control group.

## 2 Methods

### 2.1 Study design

This study was a randomised controlled trial conducted in a university community setting. The published protocol for this trial details the study design and data collection procedures (Matsumoto et al., 2025). No financial compensation was provided for participation in the trial; however, a self-help book for social anxiety was distributed to all participants at the end of the study (Matsumoto and Yoshinaga, 2024). Details regarding the updates to the study protocol are provided in the Supplemental Material (Appendix 1).

### 2.2 Ethics statement

This study was conducted in accordance with the principles of the Declaration of Helsinki. The study protocol was reviewed and approved by the Clinical Research Ethics Committee of [Blinded for Review] University Hospital on 4 March 2025 (Approval ID: 240229RIN). This trial was prospectively registered in the University Hospital Medical Information Network (UMIN) Clinical Trials Registry (ID: UMIN000057484). All participants provided written informed consent after receiving an explanation in person with printed documents.

### 2.3 Recruitment and participants

Participants were recruited from 11 universities across Japan and through a dedicated website, with recruitment commencing on 1st April 2025. Eligibility criteria included, aged 18–30 years, score ≥ 10 on the Gaze Anxiety Rating Scale Japanese version (GARS-J), ability to manage internet/email communication, and intention to remain enrolled at their university throughout the trial. The threshold was intended as a broad minimum symptom criterion to exclude participants reporting virtually no gaze-related anxiety rather than as a diagnostic cutoff. Exclusion criteria included the presence of a mental disorder, a history of suicide attempts; pregnancy, and inability to maintain contact with researchers. All participants received face-to-face study explanations and provided written informed consent. Interested individuals then completed an online registration form containing a screening questionnaire that assessed inclusion and exclusion criteria, including psychiatric history and current treatment status. When a history of psychiatric illness or relevant clinical conditions was reported, the principal investigator (K.M.) conducted a telephone interview to assess current functioning, clinical status (acute vs. remission), and eligibility. Applicants meeting diagnostic criteria for social anxiety disorder (SAD) or other psychiatric conditions were excluded and directed to university counselling services or local psychiatric clinics.

### 2.4 Interventions and control

#### 2.4.1 Bibliotherapy (Bib)

Participants allocated to the unguided bibliotherapy (Bib) group received a 206-page self-help book on gaze-related anxiety, structured into five chapters (Matsumoto and Yoshinaga, 2024). Chapter 1 provides psychoeducation on gaze-related anxiety within the context of SAD. Chapter 2 introduces Clark and Wells' (1995) cognitive model, and Chapter 3 guides users through step-by-step practice of CBT techniques for SAD. Chapter 4 presents four fictional case vignettes to illustrate the application process, and Chapter 5 prompts users to complete worksheets for relapse prevention. During the trial, participants received automated reminder emails every Monday. The reminder email indicated the chapter scheduled for that week and encouraged participants to continue reading and practising the CBT exercises.

#### 2.4.2 Digital self-help intervention (DSI)

Participants in the DSI received a web-based self-help program targeting subthreshold SAD. The program was based on a comprehensive CBT package and consisted of 10 modules with 139 slides: (1) psychoeducation and case formulation based on cognitive-behavioural models; (2) safety behaviours and self-focused attention; (3) video feedback to correct negative self-imagery; (4) attention training; (5) behavioural experiments; (6) opinion surveys; (7) reducing rumination and pre- and post-event processing; (8) processing social trauma through imagery rescripting; (9) schema work; and (10) relapse prevention. Each module included text-based materials supplemented with illustrations and figures, homework assignments, and interactive Q&A exercises, accessible at any time via computers or smartphones. Automated reminder emails were sent every Monday and Thursday. Because the DSI was designed to progress at approximately two modules per week, reminders were sent twice weekly and indicated the modules scheduled for completion.

#### 2.4.3 Waitlist control

Control participants were requested to refrain from seeking face-to-face CBT or other SAD-specific information during the trial. Following the 6-month follow-up, they were provided with the Bib intervention.

### 2.5 Outcome measures

The present trial was designed around gaze-related anxiety, and participant eligibility was defined partly on the basis of GARS-J scores. The bibliotherapy intervention was specifically framed around gaze-related anxiety, while both interventions targeted cognitive-behavioural processes relevant to social anxiety. Accordingly, the GARS-J was prespecified as the primary outcome to assess change in the symptom domain around which the trial was designed. The LSAS and SPIN were included as secondary outcomes to evaluate the generalization of these effects to broader social anxiety symptoms. Primary and secondary outcomes were assessed via online self-report at baseline, post-intervention (5 weeks), and at 3- and 6-month follow-ups.

#### 2.5.1 Primary outcome

The primary outcome was gaze-related anxiety, measured by the GARS-J, which has demonstrated high reliability and validity in Japanese university samples (see Appendix 2 in the Supplementary material). The GARS-J assesses fear and avoidance in 17 situations involving being gazed at, with total scores ranging from 0 to 102.

#### 2.5.2 Secondary outcomes

Secondary outcomes included social anxiety symptoms, measured using the Japanese version of the Liebowitz Social Anxiety Scale (LSAS) and the Social Phobia Inventory (SPIN) (Asakura et al., 2002; Nagata et al., 2013). Depressive and generalised anxiety symptoms were assessed using the Japanese version of the Patient Health Questionnaire-9 (PHQ-9) and the Generalised Anxiety Disorder-7 (GAD-7) (Muramatsu, 2014).

#### 2.5.3 Adverse events

Adverse events (AEs) and serious adverse events (SAEs) were monitored through participant self-reports throughout the trial. Any SAE was reported to the ethics committee, with an independent review committee determining the trial's continuation. Affected participants were monitored until resolution of the event.

### 2.6 Randomisation and blinding

The randomisation procedure was prespecified as part of the study design. Stratified block randomisation (block size = 15) was used according to biological sex, institution, and social anxiety severity (LSAS score < 50 vs ≥50). The allocation sequence was generated and implemented by S.H. using the randomizr package in R version 4.4.2 (R Foundation for Statistical Computing, Vienna, Austria) (Coppock, 2025). The sequence was concealed from site investigators involved in participant recruitment until assignment. S.H. contributed to the overall study design but was not involved in the development of either intervention. Owing to the nature of the interventions, participants and the principal investigator (K.M.) could not be blinded to treatment allocation after assignment. No independent outcome assessors were employed because all clinical outcomes were assessed using standardized online self-report measures. For the statistical analyses, treatment groups were masked using neutral numerical codes (“1”, “2”, and “3”), and S.H. did not have access to the code key identifying the bibliotherapy, digital self-help, and waitlist control groups during the analyses. Thus, the statistical analyses were conducted while blinded to treatment allocation, in accordance with the prespecified protocol.

### 2.7 Sample size calculation

The sample size was determined based on a power analysis for a mixed model for repeated measures, implemented using the ‘longpower’ package in R (Donohue et al., 2024). Based on a standardized effect size of 0.60 (Matsumoto et al., 2024), a total of 207 participants, 69 in each group, was estimated to provide 0.9 power. A conservative two-sided significance level of *α* = 0.025 was adopted to address the issue of multiple comparisons arising from the inclusion of two active treatment groups against a shared control.

### 2.8 Statistical analysis

Statistical analyses were conducted according to a modified intention-to-treat (mITT) principle. The mITT population included all randomised participants regardless of treatment adherence, except for seven participants who withdrew consent and requested removal of their data and one participant who was found after randomisation to have violated the eligibility criteria.

We summarised demographic data using frequencies and percentages, while clinical characteristics and baseline outcomes were described using means and standard deviations (SDs) for each group. We used one-way ANOVA to compare the mean of continuous variables and chi-squared tests to compare the proportions of categorical variables among groups. Differences in treatment adherence rates between the two intervention groups were analysed using Fisher's exact tests.

Missing data were imputed using multiple imputations of chained equations based on a fully conditional specification algorithm with the ‘mice’ package in R (van Buuren and Groothuis-Oudshoorn, 2011). Predictive mean matching was used to generate 50 imputed datasets.

We fitted linear mixed-effects models separately to each imputed dataset using the ‘lme4’ package in R (Bates et al., 2025), and combined the resulting estimates using Rubin's rules (Rubin, 1987). The models used changes from baseline at post-intervention, 3-month follow-up, and 6-month follow-up as the dependent variable. The models included time point, treatment group, their interaction, baseline score, sex, and institution as fixed effects. Participant was included as a random intercept to account for repeated observations within individuals. Model assumptions were evaluated by inspecting residual-versus-fitted plots and Q-Q plots of the conditional residuals. Estimated marginal means were used to compare each intervention group with the waitlist control at each assessment point. Dunnett's adjustment was applied to account for the two comparisons with a shared control using the ‘emmeans’ package in R (Piaskowski, 2025; Dunnett, 1955, Dunnett, 1964). Hedges' *g* was calculated by standardising the adjusted mean difference using the pooled baseline SD and applying the small-sample bias correction. The corresponding 95% CIs were obtained using the same standardisation of the confidence limits for the adjusted mean differences.

Sensitivity analyses were conducted after excluding non-adherent participants (defined as reading <2 Bib chapters or completing <5 DSI modules) and those with inconsistent or uniform response patterns, using the same multiple-imputation and mixed-effects modelling procedures as the primary analyses. To assess the potential influence of missing outcome data on the estimated treatment effects, we conducted a post hoc sensitivity analysis using delta-adjusted pattern-mixture multiple imputation under departures from the missing-at-random assumption. Further details are provided in the Supplementary material (Appendix 3).

Clinically significant outcomes were derived separately within each imputed dataset from the imputed continuous outcome scores. Treatment response was defined as a ≥ 28% reduction in LSAS score from baseline (Citrome, 2014), remission as an LSAS total score < 35 (Bandelow et al., 2006), recovery as both a SPIN total score < 19 and a PHQ-9 total score < 10 (National Collaborating Centre for Mental Health, 2024), and deterioration as a ≥ 28% increase in LSAS score from baseline (Matsumoto et al., 2024). Event proportions were estimated within each imputed dataset and combined across the 50 datasets using Rubin's rules. Risk ratios (RRs) were estimated using modified Poisson regression with robust standard errors, and odds ratios (ORs) were estimated using logistic regression; estimates and 95% CIs were combined across imputations using Rubin's rules (Rubin, 1987). For deterioration, relative risk reduction was calculated as 1 − RR. Absolute risk reduction (ARR) was defined as the risk in the waitlist control group minus that in the intervention group and was estimated within each imputed dataset using a linear probability model with robust standard errors before pooling using Rubin's rules. The number needed to treat for benefit (NNTB) was calculated as the reciprocal of the pooled ARR and is presented as a point estimate.

Statistical tests were two-sided with an α level of 0.05. Dunnett-adjusted *P* values were used for comparisons of each intervention group with the waitlist control. Statistical comparisons of baseline characteristics were conducted using SPSS version 28.0 (IBM Corp., Armonk, NY), and all other analyses were performed in R.

## 3 Results

### 3.1 Participants flow

A total of 252 participants were randomised to the Bib (*n* = 86), DSI (*n* = 83), or waitlist control (*n* = 83) groups (Fig. 1). Following allocation, seven participants withdrew their consent and requested the removal of their data. A participant in the control group was removed after undisclosed past suicide attempts and current suicidal ideation were found during the trial period (Fig. 1). Missing outcome data increased over time and were more frequent in the DSI group. For the primary outcome, missing data at post-intervention were observed in 9 of 84 participants (10.7%) in the Bib group, 24 of 80 (30.0%) in the DSI group, and 6 of 80 (7.5%) in the waitlist control group. At the six-month follow-up, the corresponding proportions were 25.0%, 35.0%, and 17.5%, respectively. Missingness by group and assessment point is detailed in the Supplementary material (Appendix 4).

**Fig. 1:**
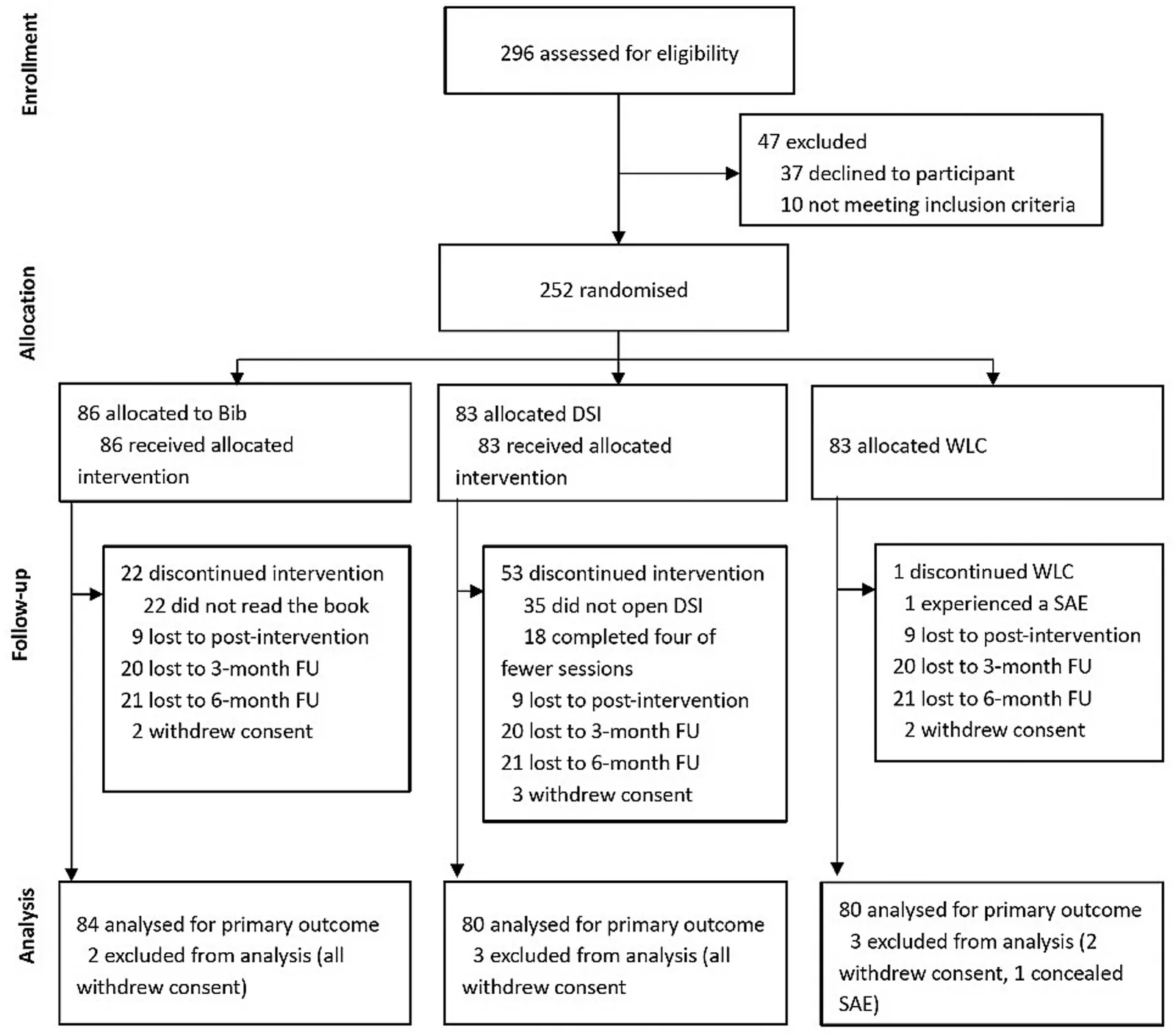
Participant flow through the trial. The flowchart illustrates the progression of participants from initial screening through the 6-month follow-up. Bib, bibliotherapy intervention; DSI, digital self-help intervention; FU, follow-up; GARS, Gaze Anxiety Rating Scale; SAE, serious adverse event; WLC, waitlist control.

### 3.2 Participant characteristics at baseline

The three groups were homogeneous at baseline regarding demographic and clinical characteristics, including age, biological sex, educational background, and residential area. Furthermore, no significant differences were observed in the baseline severity of gaze-related anxiety, social anxiety, depression, or generalised anxiety (Table 1).

**Table 1:** Demographic data and participant characteristics at baseline.

	Bib (n = 84)	DSI (n = 80)	WLC (n = 80)	*P* value
Age (years), mean (SD)	20.18 (2.01)	20.15 (2.06)	20.08 (1.98)	0.81
Biological sex, n (%)				0.60
Female	68 (81)	61 (76)	66 (81)	
Male	16 (19)	19 (24)	14 (19)	
Education, n (%)				0.75
Undergraduate	64 (76)	61 (76)	60 (75)	
Graduate	20 (24)	19 (24)	20 (25)	
Place of residence, n (%)				0.98
Tohoku	7 (8)	5 (6)	10 (12)	
Kanto	40 (48)	40 (50)	39 (48)	
Chubu	13 (15)	13 (16)	11 (14)	
Chugoku	9 (11)	6 (8)	7 (9)	
Shikoku	8 (10)	9 (11)	6 (8)	
Kyushu	7 (8)	7 (9)	7 (9)	
Clinical symptoms, mean (SD)				
GARS-J total	40.63 (14.81)	38.09 (14.42)	40.54 (14.81)	0.30
LSAS total	68.06 (24.42)	61.89 (22.56)	67.69 (24.37)	0.13
SPIN total	34.77 (14.32)	31.98 (13.30)	34.89 (13.84)	0.18
PHQ-9 total	7.74 (6.27)	7.55 (6.70)	8.40 (5.74)	0.39
GAD-7 total	5.95 (5.35)	5.30 (4.54)	6.48 (5.38)	0.16

We used one-way analysis of variance to compare the average of continuous variables and chi-square tests to compare the proportion of categorical variables among groups.

Bib, bibliotherapy intervention; DSI, digital self-help intervention; GARS-J, Japanese version of Gaze Anxiety Rating Scale; GAD-7, Generalized Anxiety Disorder-7; LSAS, Liebowitz Social Anxiety Rating Scale; PHQ-9, Patient Health Questionnaire-9; SD, standard deviation; SPIN, Social Phobia Inventory; WLC, waitlist control.

### 3.3 Adherence and engagement

Adherence rates significantly differed between the active interventions, with 70% in the Bib group compared to 38% in the DSI group (RR = 1.87, 95% CI: 1.37–2.57). In the Bib group, 45% (*n* = 38) completed all five chapters, while 28% (*n* = 22) of the DSI group completed all 10 modules. Initial engagement was also significantly higher for the Bib group (87%) than for the DSI group (56%; RR = 1.54, 95% CI: 1.25–1.91). Use of external psychological services was low and comparable across groups: 3% (*n* = 2) in the control group received psychotherapy, and 5–8% across all groups accessed general university counselling. Detailed module-by-module completion rates are provided in Supplementary material (Appendix 5).

### 3.4 Intervention effects

Pooled joint tests from the linear mixed-effects models across the 50 imputed datasets showed a significant overall effect of treatment allocation on the primary outcome, the GARS-J (*F*[2, 152.67] = 13.82, *p* < .001). The allocation × time interaction was not statistically significant (*F*[4, 206.77] = 0.71, *P* = .590). Diagnostic plots showed that the standardized conditional residuals were generally distributed around zero without substantial heteroscedasticity. Most residuals closely followed the reference line in the Q-Q plot, although some deviations were observed in the tails (see Appendix 6 in the Supplementary material). Significant overall effects of treatment allocation were also observed for the LSAS, the SPIN, and the PHQ-9, but not for the GAD-7. None of the allocation × time interactions for the secondary outcomes was statistically significant. Detailed pooled joint tests are provided in the Supplementary material (Appendix 7).

Table 2 summarises the prespecified comparisons of each intervention with the waitlist control at each assessment point. For the primary outcome, both the Bib and the DSI groups showed significantly greater reductions in the GARS-J scores than the waitlist control at all assessment points. At post-intervention, the adjusted mean differences were − 10.09 (95% CI: −14.61 to −5.56, *g* = −0.66) for the Bib and − 6.95 (95% CI: −12.11 to −1.79, *g* = −0.47) for the DSI. Significant between-group differences were maintained at the 3- and 6-month follow-ups. Summary statistics for all measures across all time points are available in the Supplementary material (Appendix 8).

**Table 2:** Adjustment mean differences in primary and secondary outcomes.

Outcome	Time	Bib vs WLC			DSI vs WLC		
		MD (95% CI)	Hedges' *g* (95% CI)	Dunnett-adjusted *p*	MD (95% CI)	Hedges' *g* (95% CI)	Dunnett-adjusted *p*
GARS-J	Post	−10.09 (−14.61, −5.56)	−0.66 (−0.96, −0.36)	<.001	−6.95 (−12.11, −1.79)	−0.47 (−0.82, −0.12)	.006
	3M	−8.02 (−12.64, −3.40)	−0.52 (−0.83, −0.22)	<.001	−6.49 (−11.71, −1.26)	−0.44 (−0.80, −0.09)	.012
	6M	−8.80 (−13.46, −4.14)	−0.58 (−0.88, −0.27)	<.001	−8.39 (−13.79, −2.99)	−0.57 (−0.94, −0.20)	.001
LSAS	Post	−13.57 (−20.86, −6.29)	−0.55 (−0.85, −0.26)	<.001	−12.47 (−20.78, −4.17)	−0.53 (−0.88, −0.18)	.002
	3M	−13.64 (−21.16, −6.12)	−0.56 (−0.86, −0.25)	<.001	−9.67 (−18.25, −1.09)	−0.41 (−0.77, −0.05)	.024
	6M	−10.50 (−18.20, −2.79)	−0.43 (−0.74, −0.11)	.005	−11.77 (−20.51, −3.03)	−0.50 (−0.87, −0.13)	.006
SPIN	Post	−6.25 (−10.42, −2.07)	−0.44 (−0.74, −0.15)	.002	−7.42 (−12.12, −2.72)	−0.54 (−0.89, −0.20)	<.001
	3M	−4.66 (−8.94, −0.38)	−0.33 (−0.63, −0.03)	.030	−4.70 (−9.52, 0.12)	−0.34 (−0.70, 0.01)	.057
	6M	−5.87 (−10.28, −1.46)	−0.41 (−0.73, −0.10)	.006	−5.63 (−10.42, −0.83)	−0.41 (−0.76, −0.06)	.018
PHQ-9	Post	−1.70 (−3.22, −0.18)	−0.28 (−0.53, −0.03)	.025	−2.61 (−4.23, −0.99)	−0.42 (−0.67, −0.16)	<.001
	3M	−0.38 (−2.02, 1.26)	−0.06 (−0.33, 0.21)	.811	−1.04 (−2.74, 0.66)	−0.17 (−0.44, 0.10)	.296
	6M	−0.97 (−2.54, 0.60)	−0.16 (−0.42, 0.10)	.290	−2.34 (−4.06, −0.63)	−0.37 (−0.65, −0.10)	.005
GAD-7	Post	−0.99 (−2.24, 0.26)	−0.18 (−0.42, 0.05)	.143	−1.53 (−2.89, −0.18)	−0.31 (−0.58, −0.04)	.023
	3M	−0.28 (−1.61, 1.05)	−0.05 (−0.30, 0.19)	.839	−0.19 (−1.63, 1.24)	−0.04 (−0.33, 0.25)	.922
	6M	−0.45 (−1.75, 0.85)	−0.08 (−0.32, 0.16)	.651	−0.35 (−1.79, 1.09)	−0.07 (−0.36, 0.22)	.796

Note: Adjusted mean differences were estimated using linear mixed-effects models with adjustment for baseline scores, sex, and institution. Hedges' g was calculated by standardising the adjusted mean difference using the pooled baseline standard deviation and applying the small-sample bias correction.

Bib, bibliotherapy intervention; CI, confidence interval; DSI, digital self-help intervention; GAD-7, Generalized Anxiety Disorder-7; GARS-J, Japanese version of Gaze Anxiety Rating Scale; LSAS, Liebowitz Social Anxiety Scale; MD, mean difference; PHQ-9, Patient Health Questionnaire-9; SPIN, Social Phobia Inventory; WLC, waitlist control; 3M, 3-month follow-up; 6M, 6-month follow-up.

For the secondary outcomes, both intervention groups showed significantly greater reductions in the LSAS scores than the waitlist control at all assessment points. For the SPIN, both interventions showed significant differences at post-intervention and the 6-month follow-up, whereas the DSI comparison at the 3-month follow-up did not reach statistical significance. For depressive symptoms, both interventions showed significant reductions at post-intervention, whereas only the DSI showed a significant difference at the 6-month follow-up. For generalised anxiety symptoms, only the DSI showed a significant difference at post-intervention.

### 3.5 Sensitivity analyses

Sensitivity analyses yielded results generally consistent with the primary analyses. Both the Bib and the DSI groups showed significantly greater reductions in the GARS-J scores than the waitlist control at all assessment points. For the LSAS, both interventions showed significantly greater reductions at post-intervention and the 6-month follow-up; however, the DSI comparison did not reach statistical significance at the 3-month follow-up (adjusted mean difference = −10.04, 95% CI: −20.91 to 0.83, Hedges' *g* = −0.41, *p* = .075). For the SPIN, both intervention groups showed significant differences at all assessment points in the sensitivity analyses. The patterns observed for depressive and generalised anxiety symptoms were also consistent with those of the primary analyses. Detailed results are provided in the Supplementary material (Appendices 9 and 10).

Post hoc missing not at random sensitivity analyses examined the potential influence of missing outcome data on the estimated treatment effects. For the primary outcome, both interventions maintained statistically significant differences from the waitlist control at all assessment points under a small departure from the missing at random assumption (*δ* = 0.20 residual SD). Under larger departures (*δ* = 0.50 and 0.80), the Bib group maintained statistically significant differences across all examined scenarios, whereas some comparisons involving the DSI group were no longer statistically significant when poorer outcomes were assumed for participants with missing data. Detailed results are presented in the Supplementary material (Appendix 11). These findings suggest that the estimated treatment effects of the DSI were relatively robust to small departures from the missing at random assumption but were sensitive to assumptions about missing outcomes under moderate to larger departures.

### 3.6 Clinically significant improvement

Fig. 2 shows the pooled estimated proportions of participants achieving clinically significant outcomes across the multiply imputed datasets. For treatment response, both the Bib and DSI groups showed higher rates than the waitlist control at post-intervention (Bib: RR = 5.51, 95% CI: 2.06 to 14.72; DSI: RR = 4.57, 95% CI: 1.65 to 12.65) and at the 6-month follow-up (Bib: RR = 3.02, 95% CI: 1.56 to 5.82; DSI: RR = 2.78, 95% CI: 1.40 to 5.56). At the 3-month follow-up, the difference was significant for Bib but not for DSI. For remission, both intervention groups showed higher rates than the waitlist control at post-intervention and the 6-month follow-up, whereas only Bib showed a significant difference at the 3-month follow-up. Recovery rates were significantly higher for DSI at post-intervention, for Bib at the 3-month follow-up, and for both intervention groups at the 6-month follow-up. Deterioration rates did not differ significantly between either intervention group and the waitlist control at any assessment point. Detailed pooled estimates, RRs, and ORs are provided in the Supplementary material (Appendix 12).

**Fig. 2:**
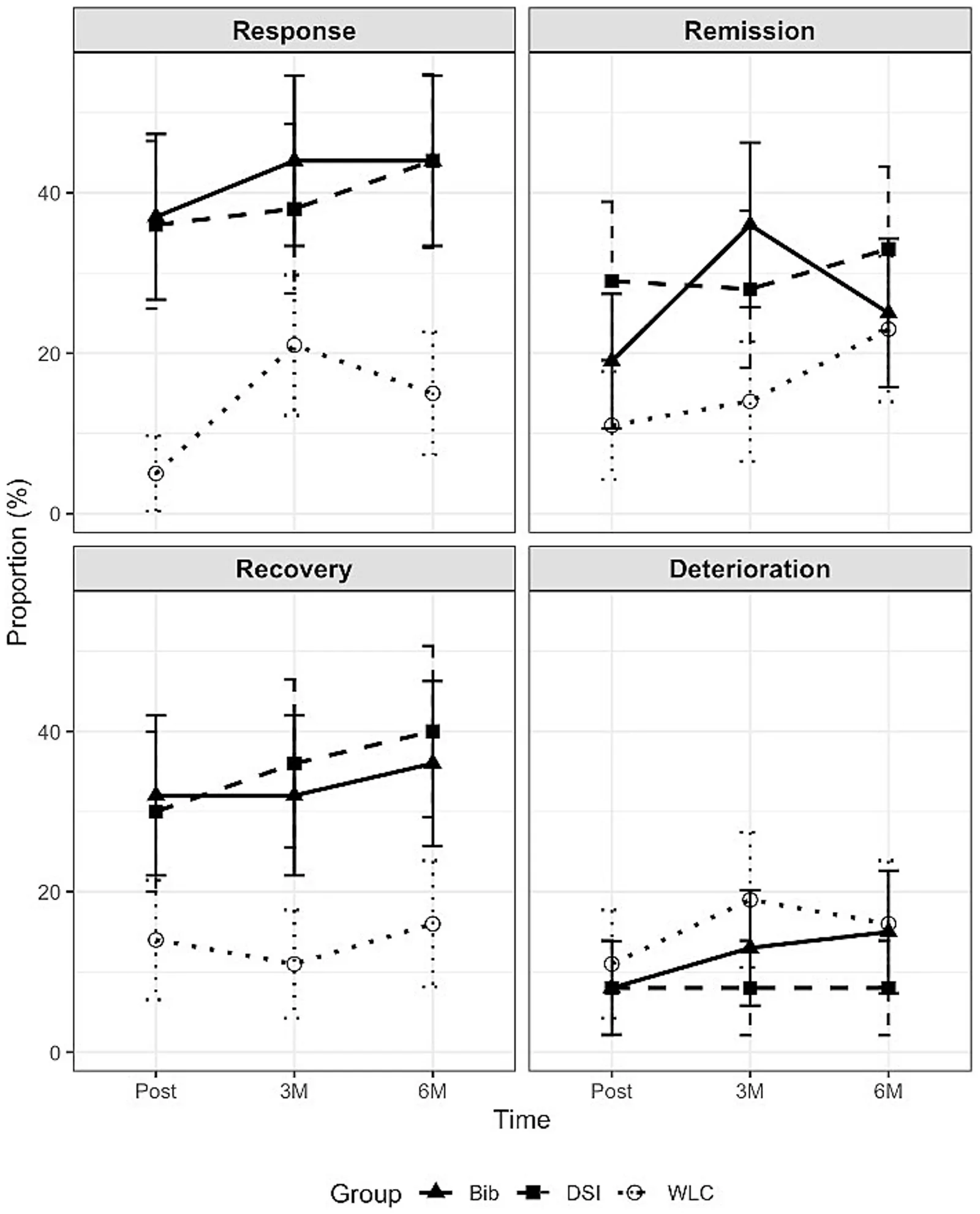
Pooled estimated proportions of participants achieving clinically significant outcomes. Points represent pooled estimated proportions across 50 multiply imputed datasets, and error bars indicate 95% confidence intervals. Response was defined as a ≥28% decrease from baseline in the Liebowitz Social Anxiety Scale (LSAS) total score; remission as an LSAS total score < 35; recovery as both a Social Phobia Inventory total score < 19 and a Patient Health Questionnaire-9 total score < 10; and deterioration as a ≥28% increase in the LSAS total score. Bib, bibliotherapy intervention; DSI, digital self-help intervention; WLC, waitlist control; 3M, 3-month follow-up; 6M, 6-month follow-up.

### 3.7 Adverse events

No SAEs related to the study interventions were reported. AEs were reported across all groups, the most common being fever (control, *n* = 5; Bib, *n* = 5; DSI, *n* = 1) and the common cold (control, *n* = 4; Bib, *n* = 3; DSI, *n* = 2). Additionally, some participants reported transient depressive symptoms (control, *n* = 3; Bib, *n* = 4; DSI, *n* = 2). As noted in the participant flow, one individual in the control group was excluded due to an undisclosed pre-existing condition identified mid-trial. A comprehensive list of all reported adverse events is provided in the Supplementary material (Appendix 13).

### 3.8 Absolute risk reduction and number needed to treat

Deterioration was numerically less frequent in the intervention groups than in the waitlist control at several assessment points; however, all 95% confidence intervals for the absolute risk reduction included zero. At the three-month follow-up, the absolute risk reduction was 10.9% (95% CI: −0.2% to 21.9%) for Bib and 9.4% (95% CI: −2.4% to 21.2%) for DSI. These results do not provide clear evidence that either intervention reduced the risk of deterioration. Detailed estimates, including exploratory NNTB point estimates, are provided in the Supplementary material (Appendix 14).

## 4 Discussion

### 4.1 Principal findings

The current multicentre randomised controlled trial evaluated two five-week self-help interventions for university students with elevated gaze-related anxiety. Both the Bib and the DSI reduced gaze-related anxiety compared with waitlist control. The effects were observed at post-intervention and remained significant at the 3- and 6-month follow-ups. Both interventions also reduced broader social anxiety symptoms. Regarding treatment adherence, the Bib group showed higher engagement than the DSI group. These findings support the effectiveness of brief, unguided interventions based on CBT for gaze-related anxiety in university students without a diagnosed mental disorder.

### 4.2 Comparisons with previous works

The present findings extend previous evidence in two important ways. First, our findings strengthen the available evidence for unguided self-help interventions for social anxiety. In the network meta-analysis by Mayo-Wilson et al. (2014), which examined treatments of SAD, unguided DSI and Bib showed standardized mean differences (SMDs) of −0.66 (95% credible interval [CrI]: −0.94 to −0.39) and − 0.84 (95% CrI: −1.08 to −0.60), respectively, compared with waitlist. The LSAS effect estimates in the present trial were smaller in magnitude (Hedges' *g* = −0.56 to −0.41), but their uncertainty intervals overlapped with those reported in the network meta-analysis. The existing evidence was based on four RCTs of DSIs (Berger et al., 2011; Titov et al., 2008, Titov et al., 2009, Titov et al., 2010) and four RCTs of Bibs reported in three publications (Rapee et al., 2007; Chung and Kwon, 2008; Furmark et al., 2009). Several trials had relatively small intervention groups (Berger et al., 2011; Titov et al., 2008), and not all directly compared unguided self-help with waitlist (Chung and Kwon, 2008; Berger et al., 2011). Some also included additional intervention components as motivational interviewing (Titov et al., 2010). Small-study effects may influence treatment estimates in meta-analyses (Sterne et al., 2011). The current multicentre randomised controlled trial investigated unguided, 5-week self-help interventions in approximately 80 participants per arm, representing one of the largest studies to date on unguided self-help for social anxiety symptoms. To our knowledge, this is the first randomised controlled trial to demonstrate the effectiveness of self-help interventions for gaze-related anxiety.

### 4.3 Treatment engagement

Although full program completion was limited, 70% of Bib participants read at least two chapters, and 38% of DSI participants completed at least five modules, both meeting our predefined adherence criteria. In previous trials evaluating unguided DSIs for social anxiety, completion rates ranged from 33% to 68% (Titov et al., 2008, Titov et al., 2009, Titov et al., 2010; Matsumoto et al., 2024), though adherence definitions varied across studies. Regarding bibliotherapy for social anxiety, Rapee et al. (2007) reported a mean of 4.11 chapters read, whereas Furmark et al. (2009) reported a mean completion of 5.86 out of nine chapters. Although adherence definitions varied across studies—warranting caution when making direct comparisons—roughly half of participants engaging in Bib or DSI appear to engage deeply enough to meet the threshold for meaningful CBT practice.

In a previous trial evaluating the same DSI over a 10-week period, 65% of participants completed the program (Matsumoto et al., 2024). Unlike the present study, that trial offered financial compensation (5000 JPY; approximately 32 USD) for completing research assessments. By contrast, the present trial provided no monetary incentives, though participants received a complimentary self-help book (1500 JPY; approximately 9.50 USD) upon study completion (Matsumoto and Yoshinaga, 2024). Monetary incentives may encourage effortful behaviour (Medvedev et al., 2024). Future studies could examine whether incentive strategies improve engagement with unguided CBT among university students.

In the current trial, both interventions were based on the Clark and Wells (1995) model. In the Bib intervention, Chapters 1 and 2 introduced psychoeducation and the cognitive-behavioural model of social anxiety, respectively, while Chapter 3 provided specific CBT techniques and step-by-step guidance for independent practice (Matsumoto and Yoshinaga, 2024). In the DSI intervention, the first five modules introduced core CBT techniques, including reducing safety behaviours, attention training, and behavioural experiments. A recent factorial trial of internet-based CBT for SAD found that psychoeducation and exposure contributed to greater symptom improvement at post-treatment (Šipka et al., 2025). Both interventions in the present trial introduced psychoeducation during the first week and provided exposure-based behavioural exercises by the third week. Participants who discontinued the interventions early during the trial may nevertheless have benefited from exposure to the cognitive-behavioural model and techniques introduced in the initial stages. Future studies should investigate the optimal amount and frequency of content, along with the most effective sequencing of established CBT components (Clark and Wells, 1995), to improve adherence and optimize outcomes in unguided self-help interventions.

### 4.4 Strength and limitations

The present trial has several methodological strengths. First, this study was designed a multicenter, three-arm randomised controlled trial comparing with a waitlist control group. The sample size surpassed the predefined target of 69 participants per arm. Second, the 5-week intervention included a 6-month follow-up. Third, we evaluated gaze-related anxiety alongside social anxiety symptoms and clinically meaningful metrics, such as treatment response, remission, recovery, and deterioration. Finally, the statistical analyses employed multiple imputation, mixed-effects models and the sensitivity analyses, ensuring the statistical rigor and robustness of the primary findings.

Several limitations warrant consideration. First, participants were self-selected university students, predominantly female, restricting the generalizability of our findings to broader clinical populations. Additionally, the absence of structured diagnostic interviews or standardized functional impairment tools means some participants may have met criteria for SAD, precluding definitive confirmation of subthreshold status. Second, the waitlist control design did not account for treatment expectancy or nonspecific therapeutic effects. Third, because Bib and DSI differed in content and structure despite sharing a theoretical framework, outcome differences between the two modalities cannot be attributed exclusively to the delivery format. Fourth, missing outcome data accumulated over time, particularly within the DSI group. Although multiple imputation addressed this attrition, the primary analyses assumed data were missing at random; thus, bias due to non-random missingness cannot be ruled out. Finally, the lack of participant blinding, reliance on self-report measures, and the principal investigator's authorship of the Bib material may have introduced performance, reporting, or investigator bias.

### 4.5 Future directions and implications

The present findings should be considered in the context of existing clinical guidelines for SAD. Although individual CBT is an established treatment for SAD (Carpenter et al., 2018), barriers to accessing and sustaining psychological treatment remain, particularly among young people and individuals outside clinical services (Pilling et al., 2013). The NICE guideline also highlights the need for research to improve access to and engagement with effective psychological interventions (NICE, 2013). In this regard, our findings suggest that brief, fully unguided self-help based on CBT may offer an accessible, low-intensity intervention for university students experiencing gaze-related and broader social anxiety symptoms. Clinically significant improvements were observed within five weeks, although not all participants achieved treatment response and engagement, particularly with the DSI, remained limited. These findings support further investigation of unguided self-help as a potential component of early intervention and stepped-care approaches, rather than as a substitute for established treatments for SAD. Future pragmatic trials should examine its acceptability, effectiveness, and implementation in university settings.

## 5 Conclusions

Brief, fully unguided self-help interventions based on CBT delivered through either Bib or a DSI programme reduced gaze-related and broader social anxiety symptoms among university students. These findings support brief self-help as a potentially accessible and scalable low-intensity option, although further work is needed to optimize engagement and clarify its role within stepped-care services.

## CRediT authorship contribution statement

KM and MN conceived of the study and designed the trial protocol. KM managed and administered the overall trial and was responsible for funding acquisition. SH contributed to the implementation of the randomised allocation, performed the statistical analysis, acquired funding, and drafted the manuscript. All authors contributed to the participant recruitment, data collection, and critical revision of the manuscript for important intellectual content. All authors have read and approved the final version for publication and agree to be accountable for all aspects of this work.

## Transparency declaration

The lead author and manuscript guarantor affirms that the manuscript is an honest, accurate, and transparent account of the study being reported; that no important aspects of the study have been omitted; and that any discrepancies from the study as planned and registered have been explained.

## Declaration of Generative AI and AI-assisted technologies in the writing process

The authors made use of Gemini (Google) to assist with the language editing and proofreading of this article to improve clarity and expression. After using this tool, the authors reviewed and edited the content as needed and take full responsibility for the content of the publication.

## Funding

This work was supported by the 2025 Clinical Research Grant for Co-medical Staff from the Foundation for Clinical Research Promotion (Grant No. 25-Comed-Clin-No3), the Young Researchers Support Programme at 10.13039/100008702Kagoshima University, Basic Educational and Research Funds from Kagoshima University, and 10.13039/100009619AMED under Grant Numbers JP256f0137012j0001 and JP266f0137012j0002. This work was supported by JSPS KAKENHI Grant Number JP25K00195. The funders had no role in the study design, data collection, data analysis, or decision to submit this manuscript for publication.

## Declaration of competing interest

KM is the author of the self-help book used in this intervention. The other authors declare no competing interests.

## Data Availability

In the Supplementary Data 1, dataset that support the findings of this study are openly available in figshare at: Matsumoto K. Datasets for Primary and Sensitivity Analyses from an RCT on Book-based and Internet-based CBT for Gaze Anxiety. *figshare.* Last edited on 2026-1-20. DOI: doi:https://doi.org/10.6084/m9.figshare.31095040. The R code used for multiple imputation of missing data and the primary statistical analyses is provided in the Supplementary material (Appendix 15).
