# Supplementary material for: Unguided bibliotherapy and digital self-help interventions based on cognitive behavioural therapy for university students with gaze-related anxiety and social anxiety symptoms: a randomised controlled trial
Source: Internet Interv. 2026 Nov 6;46:101023. doi: 10.1016/j.invent.2026.101023 (PMC13761239; doi:10.1016/j.invent.2026.101023)
Supplement: Supplementary file 1 [file mmc1.docx]

**Supplementary Material**

**Unguided bibliotherapy and digital self-help interventions based on cognitive behavioural therapy for university students with gaze-related anxiety and social anxiety symptoms: a randomised controlled trial**

Kazuki Matsumoto, Sayo Hamatani, Yoshihiko Kunisato, Katsunori Watanabe, Shinobu Nagata, Akiko Maeno, Mizue Yokoo, Yasuhiro Kimura, Naomi Sunami, Masatoshi Ikeda, Makiko Kasai, Rio Kamashita, Yoshifumi Mizuno, Masayuki Nakamura

Index of Contents

Appendix 1: Update of the study protocol	4

Sample size calculation procedures	4

Statistical software	4

Modified intention-to-treat analysis	4

Recovery as a clinically significant improvement indicator	4

Statistical analysis of treatment adherence	4

Statistical analyses revised during peer review	4

*Multiple imputation and pooling of estimates.*	4

*Specification of institution in the mixed-effects models*	5

*Calculation of standardised effect sizes*	5

*Analysis of clinically significant outcomes*	5

*Post hoc sensitivity analysis for missing outcome data*	6

Appendix 2: Validity and reliability of the GARS-J	6

Data Cleaning	6

Reliability Test	6

Validity Test	7

Test-Retest Reliability	7

Appendix 3: Methods of post hoc missing not at random sensitivity analysis	8

Appendix 4: Missing outcome data	9

Table S1. Missing outcome data for the outcomes by treatment group and assessment point	9

Appendix 5: Detailed module-by-module completion rates	10

Appendix 6: Assessment of model assumptions	10

Model assumptions were assessed across all 50 imputed datasets by inspecting residuals-versus-fitted plots and normal Q-Q plots of the standardized conditional residuals. The diagnostic patterns were generally similar across imputations. Representative plots from the first imputed dataset are shown below.	10

The Residuals-Versus-Fitted Plot	10

The Q-Q Plot of Conditional Residuals	11

Appendix 7: Pooled joint tests from the linear mixed-effects models for secondary outcomes	12

Table S2. Pooled joint tests from the linear mixed-effects models	12

Appendix 8: Summary statistics	13

Table S3. All outcome measures across all time point	13

Appendix 9: Results of sensitivity analyses for adherence	14

Appendix 10: Adjustment mean difference in sensitivity analyses	15

Table S4. Adjustment mean differences in sensitivity analyses	15

Appendix 11: Results of sensitivity analyses for missing not at random	16

Table S5. Sensitivity analysis assuming poorer outcomes among participants with missing data in the Bib group only	16

Table S6. Sensitivity analysis assuming poorer outcomes among participants with missing data in the DSI group only	17

Table S7. Sensitivity analysis assuming better outcomes among participants with missing data in the waitlist control group only	19

Table S8. Sensitivity analysis assuming poorer outcomes among participants with missing data in both intervention groups	20

Appendix 12: Details of dichotomous outcomes	23

Table S9. Pooled proportions and risk ratios for clinically significant outcomes relative to the waitlist control	23

Table S10. Pooled proportions and odds ratios for clinically significant outcomes relative to the waitlist control	24

Figure S1. Sensitivity of adjusted treatment effects on gaze-related anxiety to alternative assumptions about missing data	25

Figure S2. Sensitivity of adjusted treatment effects on the Liebowitz Social Anxiety Scale to alternative assumptions about missing data	26

Appendix 13: All adverse events each group	30

Table S11. Adverse event and serious adverse events during this trial	30

Appendix 14: Details of absolute risk reduction and number needed to treat analyses	31

Table S12. Results of Deterioration risk analyses	31

Appendix 15: Complete R code for the primary analyses	32

Reference	97

# **Appendix 1:** **Update of the study protocol**

## Sample size calculation procedures

The sample size calculation was updated from a multiple regression analysis using G*Power (version 3.1.9.7; The G*Power Team) to a mixed model for repeated measures power analysis, using the 'longpower' package in R (version 4.4.2; R Foundation for Statistical Computing, Vienna, Austria; Donohue, Edland, & Hu, 2024).

## Statistical software

Although we originally planned to use SPSS (IBM Corp., Armonk, NY, USA), statistical analyses were conducted using R too. This ensured consistency across our methods, as the version of SPSS available to us did not support the Dunnett’s tests required for our mixed models.

## Modified intention-to-treat analysis

Although the original protocol stated that analyses would follow the intention-to-treat principle, we performed a modified intention-to-treat analysis. This update was necessary because seven participants withdrew their consent and requested that their data not be used, and one participant was excluded after registration for concealing information that violated the eligibility criteria.

## Recovery as a clinically significant improvement indicator

In addition to the predefined indicators of treatment response, remission, and deterioration outlined in the protocol (Matsumoto et al., 2025), we also included recovery as a clinically significant improvement indicator.

## Statistical analysis of treatment adherence

The protocol was updated to include Fisher’s exact test. This was employed to examine the differences in engagement rates—specifically, the proportion of participants who utilised the self-help materials as intended—between the two intervention groups.

## Statistical analyses revised during peer review

### *Multiple imputation and pooling of estimates.*

During revision, we identified that although multiple imputation had been performed for missing outcome data, the analyses reported in the original submission had inadvertently been based on a single completed imputed dataset rather than estimates combined across multiple imputed datasets. We therefore repeated the primary and secondary continuous outcome analyses using 50 imputed datasets generated by predictive mean matching. The same linear mixed-effects model was fitted separately to each imputed dataset, and the resulting estimates were combined using Rubin’s rules. The sensitivity analyses were repeated using the same procedure.

### *Specification of institution in the mixed-effects models*

Institution was included as a fixed covariate in the revised mixed-effects models. During revision, we also examined a model including institution as a random intercept; however, this specification frequently resulted in singular fits because the estimated between-institution variance was approximately zero for several outcomes. Institution was therefore retained as a fixed effect, while participant was included as a random intercept to account for repeated observations within individuals.

### *Calculation of standardised effect sizes*

In the original submission, the adjusted mean differences and Hedges’ g values in Table 2 were derived from different quantities: the adjusted mean differences were obtained from the mixed-effects models, whereas Hedges’ g was calculated from observed change scores. To ensure that both estimates referred to the same treatment contrast, Hedges’ g was recalculated by standardising the adjusted mean difference from the mixed-effects model using the pooled baseline SD and applying the small-sample bias correction. The corresponding 95% confidence interval (CI)s were recalculated using the same standardisation.

### *Analysis of clinically significant outcomes*

The original protocol specified Fisher’s exact tests for treatment response, remission, and deterioration. However, Fisher’s exact test does not provide estimates that can be directly combined across multiply imputed datasets using Rubin’s rules. In the revised analyses, clinically significant outcomes were therefore derived separately within each of the 50 imputed datasets from the imputed continuous outcome scores. Risk ratios were estimated using modified Poisson regression with robust standard errors, and odds ratios were estimated using logistic regression. Estimates were then combined across imputations using Rubin’s rules. Pooled event proportions were calculated using the same multiple-imputation framework. For deterioration, absolute risk reduction was estimated within each imputed dataset using a linear probability model with robust standard errors and then pooled using Rubin’s rules. The number needed to treat for benefit was calculated from the pooled absolute risk reduction. These revisions did not change the predefined primary outcome, assessment points, or intervention groups, and were made to ensure that missing-data uncertainty was appropriately incorporated into the statistical estimates.

### *Post hoc sensitivity analysis for missing outcome data*

Following receipt of the first-round peer review comments, we conducted an additional post hoc sensitivity analysis to assess the robustness of the treatment effect estimates to departures from the missing-at-random assumption. This analysis was not prespecified in the original protocol and was introduced in response to the first-round peer review to evaluate the potential influence of missing outcome data on the study findings. Detailed methods and results are provided in Appendices 3 and 11, respectively.

# **Appendix 2: Validity and reliability of the GARS-J**

To assess the psychometric properties of the Japanese version of the Gaze Anxiety Rating Scale (GARS-J) (Matsumoto et al., 2025), we conducted validation and reliability analyses using data obtained during the eligibility screening and baseline assessment phase of the randomized controlled trial. Although 252 university students were ultimately enrolled in the trial, a total of 262 students completed the GARS-J during the screening process and were therefore included in the psychometric analyses presented in this appendix.

## Data Cleaning

Data cleaning was performed prior to the analyses. We initially examined careless responding by identifying participants who provided invariant responses across all GARS-J items (e.g., selecting the same response option for every item); no participants met this criterion. Multivariate outliers were then identified using Mahalanobis distance based on responses to all GARS-J items. Using a significance threshold of *p* < 0.001, 11 participants were identified as multivariate outliers and excluded from subsequent validation analyses.

## Reliability Test

After excluding the 11 multivariate outliers, data from 251 participants were used for reliability analyses. The GARS-J was hypothesized to comprise two factors: “Anxiety/Fear” and “Avoidance.” Internal consistency was assessed using Cronbach’s alpha with the ‘psych’ package in R (Revelle, 2026). The analysis yielded alpha coefficients of 0.89 for Anxiety/Fear and 0.85 for Avoidance. Given that alpha coefficients above 0.80 generally indicate good internal consistency, these findings suggest that the GARS-J provides stable and reliable measurements.

## Validity Test

Construct validity was examined using confirmatory factor analysis (CFA) based on data from the 251 participants retained after data cleaning. The CFA tested the hypothesized two-factor structure consisting of Anxiety/Fear and Avoidance. The model included correlated residuals between anxiety and avoidance ratings within the same social situations, as well as several additional residual correlations reflecting similarities between items. Model fit was evaluated using robust maximum likelihood estimation with the ‘lavaan’ package in R (Rosseel, 2012). The results demonstrated acceptable model fit (robust Comparative Fit Index [CFI] = 0.90, robust Tucker-Lewis Index [TLI] = 0.89, and Root Mean Square Error of Approximation [RMSEA] = 0.06). Although the TLI was slightly below the conventional cutoff of 0.90, the overall pattern of fit indices supported the adequacy of the hypothesized two-factor structure. These findings support the construct validity of the GARS-J as a measure of gaze-related social anxiety.

## Test-Retest Reliability

Temporal stability was examined in 131 participants who completed the GARS-J again after a one-week interval. The results showed a Pearson correlation coefficient of *r* = 0.80 (95% CI: 0.73 to 0.85, *p* < 0.001) and an intraclass correlation coefficient of ICC (3,1) = 0.80 (95% CI: 0.72 to 0.85). Although a small but statistically significant difference was observed between the first and second assessments (mean difference = −2.16, 95% CI: −3.81 to −0.52, *p* = 0.01), the magnitude of the correlations indicates that the GARS-J possesses adequate temporal stability for use as an outcome measure in clinical research.

# **Appendix 3: Methods of post hoc missing not at random sensitivity analysis**

To assess the potential influence of missing outcome data on the estimated treatment effects, we conducted a post hoc sensitivity analysis using a delta-adjusted pattern-mixture multiple-imputation approach, following Goldberg et al. (2021). The analysis included the primary outcome (GARS-J) and four secondary outcomes (LSAS, SPIN, PHQ-9, and GAD-7) at post-intervention and the 3- and 6-month follow-ups.

The primary analyses assumed that outcome data were missing at random (MAR). To examine departures from this assumption, only originally missing follow-up scores were shifted within each of the 50 multiply imputed datasets. Following Goldberg et al. (2021), we specified δ values of 0.20, 0.50, and 0.80, representing small, moderate, and large hypothetical departures from MAR, respectively, based on conventional standardised effect sizes (Cohen, 1988). Higher scores indicated greater symptom severity for all outcomes.

Four scenarios were examined: (1) poorer outcomes among participants with missing data in the Bib group; (2) poorer outcomes among participants with missing data in the DSI group; (3) poorer outcomes among participants with missing data in both intervention groups; and (4) better outcomes among participants with missing data in the waitlist control group. These scenarios were designed to examine assumptions potentially unfavourable to the estimated intervention effects. Observed values were left unchanged, and adjusted imputed scores were restricted to the theoretical ranges of their respective instruments.

For each scenario, the same linear mixed-effects models and multiple-imputation pooling procedures as those used in the primary analyses were applied, with Dunnett-adjusted comparisons against the waitlist control. The MAR analysis (δ = 0) served as the reference. The complete R code is provided in Appendix 15.

These analyses were exploratory. The specified δ values represent hypothetical deviations in unobserved outcomes rather than observed symptom deterioration or clinically established thresholds.

# **Appendix 4: Missing outcome data**

## Table S1. Missing outcome data for the outcomes by treatment group and assessment point

| Outcome/Assessment | Bib (n = 84) | DSI (n = 80) | Waitlist control (n = 80) |
| --- | --- | --- | --- |
| GARS |  |  |  |
| Post | 9 (10.7%) | 24 (30.0%) | 6 (7.5%) |
| 3M | 18 (21.4%) | 26 (32.5%) | 9 (11.3%) |
| 6M | 21 (25.0%) | 28 (35.0% | 14 (17.5% |
| LSAS |  |  |  |
| Post | 9 (10.7%) | 23 (28.75%) | 6 (7.5%) |
| 3M | 18 (21.4%) | 26 (32.5%) | 9 (11.3%) |
| 6M | 21 (25.0%) | 28 (35.0% | 14 (17.5% |
| SPIN |  |  |  |
| Post | 9 (10.7%) | 23 (28.75%) | 6 (7.5%) |
| 3M | 18 (21.4%) | 26 (32.5%) | 9 (11.3%) |
| 6M | 21 (25.0%) | 28 (35.0% | 15 (18.75%) |
| PHQ-9 |  |  |  |
| Post | 9 (10.7%) | 23 (28.75%) | 6 (7.5%) |
| 3M | 18 (21.4%) | 26 (32.5%) | 9 (11.3%) |
| 6M | 21 (25.0%) | 28 (35.0% | 14 (17.5% |
| GAD-7 |  |  |  |
| Post | 9 (10.7%) | 23 (28.75%) | 6 (7.5%) |
| 3M | 18 (21.4%) | 26 (32.5%) | 9 (11.3%) |
| 6M | 21 (25.0%) | 28 (35.0% | 14 (17.5% |

*Note. Values are number of missing observations within the modified intent-to-treat population. Missingness was calculated before multiple imputation.*

Bib, bibliotherapy intervention; DSI, digital self-help intervention; SD, standard deviation; 3M, three-month follow-up; 6M, six-month follow-up.

# **Appendix 5: Detailed module-by-module completion rates**

In the bibliotherapy (Bib) group, 38 (45%) completed all five chapters of the book. Fifty-three (63%) completed at least four chapters, 55 (65%) completed at least three, 59 (70%) read at least two chapters, and 73 (87%) read the first chapter. The remaining 11 participants (13%) did not start reading the book. In the digital self-help intervention (DSI), 22 (28%) completed all 10 sessions of the programme. Thirty (38%) completed at least five sessions. Forty-five (56%) initiated the programme by accessing the DSI platform and completing at least one module. Thirty-five (44%) never accessed the DSI programme. In the control group, two participants (3%) received psychotherapy (person-centered approach and focusing). General counseling sessions at university counselling centers were used by six participants (7%) in the Bib group, four participants (5%) in the DSI group, and six participants (8%) in the control group.

# **Appendix 6: Assessment of model assumptions**

## Model assumptions were assessed across all 50 imputed datasets by inspecting residuals-versus-fitted plots and normal Q-Q plots of the standardized conditional residuals. The diagnostic patterns were generally similar across imputations. Representative plots from the first imputed dataset are shown below.

## The Residuals-Versus-Fitted Plot

The standardized conditional residuals were generally distributed around zero, with no pronounced funnel-shaped pattern. A slight trend was observed across fitted values, but there was no evidence of substantial heteroscedasticity.

Residuals-versus-fitted plot. Standardized conditional residuals from the GARS-J linear mixed-effects model were plotted against conditional fitted values. The plot shown is based on the first of 50 imputed datasets and is presented as a representative diagnostic plot.

## The Q-Q Plot of Conditional Residuals

The majority of standardized conditional residuals closely followed the diagonal reference line. Some deviations were observed in both tails, particularly in the upper tail, indicating mild departures from normality. Overall, no substantial violation of the distributional assumptions was evident.

Normal Q-Q plot of standardized conditional residuals from the GARS-J linear mixed-effects model. The plot shown is based on the first of 50 imputed datasets and is presented as a representative diagnostic plot.

# **Appendix 7: Pooled joint tests from the linear mixed-effects models for secondary outcomes**

Significant overall effects of treatment allocation were observed for LSAS, SPIN, and PHQ-9, but not GAD-7. None of the allocation × time interactions was statistically significant. Significant overall effects of time were observed for all secondary outcomes.

## Table S2. Pooled joint tests from the linear mixed-effects models

| **Outcome** | **Effect** | **df_1_** | **df_2_** | ***F*** | ***p*** |
| --- | --- | --- | --- | --- | --- |
| GARS-J |  |  |  |  |  |
|  | Treatment allocation | 2 | 153.59 | 13.82 | <.001 |
|  | Time | 2 | 226.46 | 1.15 | .320 |
|  | Allocation × Time | 4 | 206.77 | 0.71 | .590 |
| LSAS |  |  |  |  |  |
|  | Treatment allocation | 2 | 153.59 | 11.19 | <.001 |
|  | Time | 2 | 199.35 | 4.04 | .019 |
|  | Allocation × Time | 4 | 198.23 | 0.76 | .553 |
| SPIN |  |  |  |  |  |
|  | Treatment allocation | 2 | 166.55 | 7.74 | <.001 |
|  | Time | 2 | 193.98 | 4.39 | .014 |
|  | Allocation × Time | 4 | 212.52 | 0.58 | .678 |
| PHQ-9 |  |  |  |  |  |
|  | Treatment allocation | 2 | 181.17 | 6.23 | .002 |
|  | Time | 2 | 236.25 | 5.38 | .005 |
|  | Allocation × Time | 4 | 242.81 | 1.16 | .329 |
| GAD-7 |  |  |  |  |  |
|  | Treatment allocation | 2 | 164.48 | 1.35 | .262 |
|  | Time | 2 | 198.86 | 15.08 | <.001 |
|  | Allocation × Time | 4 | 195.87 | 1.00 | .411 |

Tests were obtained from linear mixed-effects models fitted separately to each of 50 multiply imputed datasets and combined using multiple-imputation pooling procedures. Models included time point, treatment group, their interaction, baseline score, sex, and institution as fixed effects, with participant as a random intercept.

# **Appendix 8: Summary statistics**

## Table S3. All outcome measures across all time point

| Outcome | Waitlist control | Bib | DSI |
| --- | --- | --- | --- |
| GARS-J, mean (SD) |  |  |  |
| Pre | 40.54 (14.81) | 40.63 (15.59) | 38.09 (14.42) |
| Post | 42.31 (15.46) | 31.21 (14.66) | 34.11 (16.81) |
| 3M | 40.59 (16.60) | 30.76 (16.43) | 31.11 (15.34) |
| 6M | 41.15 (14.83) | 31.62 (16.60) | 29.67 (13.85) |
| LSAS, mean (SD) |  |  |  |
| Pre | 67.69 (24.37) | 68.06 (24.42) | 61.89 (22.56) |
| Post | 69.45 (26.70) | 54.32 (24.31) | 52.96 (24.97) |
| 3M | 65.23 (27.33) | 49.47 (27.43) | 50.48 (25.83) |
| 6M | 64.59 (25.09) | 53.94 (28.48) | 47.71 (24.71) |
| SPIN, mean (SD) |  |  |  |
| Pre | 34.89 (13.84) | 34.77 (14.32) | 31.98 (13.30) |
| Post | 34.76 (14.56) | 27.43 (13.25) | 25.82 (14.23) |
| 3M | 31.72 (13.83) | 25.08 (15.93) | 25.04 (14.23) |
| 6M | 31.88 (13.82) | 25.71 (15.45) | 24.23 (13.32) |
| PHQ-9, mean (SD) |  |  |  |
| Pre | 8.40 (5.74) | 7.74 (6.27) | 7.55 (6.70) |
| Post | 8.20 (5.49) | 6.09 (5.11) | 5.37 (4.28) |
| 3M | 6.16 (5.17) | 5.23 (4.47) | 4.74 (4.10) |
| 6M | 7.36 (5.89) | 5.94 (5.43) | 4.37 (3.20) |
| GAD-7, mean (SD) |  |  |  |
| Pre | 6.42 (5.38) | 5.95 (5.35) | 5.30 (4.54) |
| Post | 6.18 (5.13) | 4.79 (4.47) | 4.32 (3.53) |
| 3M | 3.89 (4.11) | 3.14 (4.00) | 3.17 (3.58) |
| 6M | 5.02 (4.88) | 4.16 (4.69) | 3.73 (3.89) |

*Note. Descriptive statistics are based on observed data before multiple imputation. The numbers of observed participants were WLC: 80 at baseline, 74 at post-intervention, 71 at 3 months, and 66 at 6 months; Bib: 84, 75, 66, and 63, respectively; and DSI: 80, 57, 54, and 52, respectively. Exceptions were GARS-J at post-intervention in the DSI group (n = 56) and SPIN at 6 months in the WLC group (n = 65). Missing outcome data were handled using multiple imputation in the inferential analyses.*

Bib, bibliotherapy intervention; DSI, digital self-help intervention; SD, standard deviation; 3M, three-month follow-up; 6M, six-month follow-up.

# **Appendix 9: Results of sensitivity analyses for adherence**

Sensitivity analyses were conducted after excluding non-adherent participants and those with inconsistent or uniform response patterns, using the same multiple-imputation and mixed-effects modelling procedures as the primary analyses. The allocation × time interaction was not statistically significant for GARS-J (*F*[4, 240.49] = 1.34, *p* = .255), LSAS (*F*[4, 236.38] = 0.46, *p* = .762), SPIN (*F*[4, 256.48] = 1.61, *p* = .171), PHQ-9 (*F*[4, 259.46] = 1.19, *p* = .315), or GAD-7 (*F*[4, 242.89] = 1.01, *p* = .404). The findings were generally consistent with the primary analyses. For GARS-J, both the Bib and DSI groups showed significantly greater reductions than the waitlist control at post-intervention and at the 3- and 6-month follow-ups. For LSAS, both interventions showed significantly greater reductions at post-intervention and the 6-month follow-up; at the 3-month follow-up, the difference remained significant for Bib, whereas the DSI comparison did not reach statistical significance (*p* = .075). For SPIN, both intervention groups showed significantly greater reductions than the waitlist control at all assessment points. For PHQ-9, both groups showed significant reductions at post-intervention, while only DSI showed a significant difference at the 6-month follow-up. For GAD-7, only DSI showed a significant difference at post-intervention.

# **Appendix 10: Adjustment mean difference in sensitivity analyses**

## Table S4. Adjustment mean differences in sensitivity analyses

| Outcome | Time | Bib vs WLC | | | DSI vs WLC | | |
| --- | --- | --- | --- | --- | --- | --- | --- |
|  |  | MD (95% CI) | Hedges’ *g* (95% CI) | *Dunnett-adjusted p* | MD (95% CI) | Hedges’ *g* (95% CI) | *Dunnett-adjusted p* |
| GARS-J |  |  |  |  |  |  |  |
|  | Post | −11.20 (−16.05, −6.34) | −0.78 (−1.12, −0.44) | <.001 | −10.95 (−17.23, −4.67) | −0.72 (−1.14, −0.31) | <.001 |
|  | 3M | −8.36 (−13.32, −3.40) | −0.58 (−0.93, −0.24) | <.001 | −6.68 (−13.08, −0.28) | −0.44 (−0.86, −0.02) | .039 |
|  | 6M | −9.52 (−14.55, −4.48) | −0.66 (−1.01, −0.31) | <.001 | −10.32 (−16.75, −3.88) | −0.68 (−1.11, −0.26) | <.001 |
| LSAS |  |  |  |  |  |  |  |
|  | Post | −14.22 (−22.34, −6.10) | −0.60 (−0.95, −0.26) | <.001 | −15.34 (−25.87, −4.81) | −0.63 (−1.06, −0.20) | .003 |
|  | 3M | −12.89 (−21.13, −4.65) | −0.55 (−0.90, −0.20) | .001 | −10.04 (−20.91, 0.83) | −0.41 (−0.86, 0.03) | .075 |
|  | 6M | −13.44 (−21.92, −4.96) | −0.57 (−0.93, −0.21) | <.001 | −12.01 (−22.88, −1.14) | −0.49 (−0.94, -0.05) | .027 |
| SPIN |  |  |  |  |  |  |  |
|  | Post | −7.32 (−11.95, −2.69) | −0.55 (−0.89, −0.20) | <.001 | −11.50 (−17.44, −5.57) | −0.84 (−1.27, −0.40) | <.001 |
|  | 3M | −5.23 (−9.94, −0.51) | −0.39 (−0.74, −0.04) | .027 | −6.69 (−12.86, −0.52) | −0.49 (−0.93, −0.04) | .031 |
|  | 6M | −7.71 (−12.55, −2.87) | −0.57 (−0.93, −0.21) | <.001 | −8.06 (−14.12, −1.99) | −0.58 (−1.03, −0.14) | .006 |
| PHQ-9 |  |  |  |  |  |  |  |
|  | Post | −2.05 (−3.67, −0.43) | −0.35 (−0.63, −0.07) | .010 | −2.62 (−4.67, −0.58) | −0.47 (−0.83, −0.10) | .009 |
|  | 3M | −0.25 (−1.91, 1.40) | −0.04 (−0.33, 0.24) | .905 | −1.24 (−3.33, 0.84) | −0.22 (−0.59, 0.15) | .316 |
|  | 6M | −1.07 (−2.76, 0.62) | −0.18 (−0.47, 0.11) | .275 | −2.19 (−4.29, −0.08) | −0.39 (−0.76, −0.02) | .040 |
| GAD-7 |  |  |  |  |  |  |  |
|  | Post | −1.23 (−2.67, 0.21) | −0.24 (−0.51, 0.04) | .105 | −1.86 (−3.67, −0.05) | −0.35 (−0.69, −0.01) | .043 |
|  | 3M | −0.51 (−1.96, 0.94) | −0.10 (−0.38, 0.18) | .648 | −0.58 (−2.41, 1.25) | −0.11 (−0.46, 0.24) | .694 |
|  | 6M | −0.80 (−2.31, 0.70) | −0.15 (−0.45, 0.14) | .390 | −0.21 (−2.11, 1.68) | −0.04 (−0.40, 0.32) | .942 |

*Note. Adjusted mean differences were estimated using linear mixed-effects models with adjustment for baseline scores, sex, and institution. Hedges’ g was calculated by standardising the adjusted mean difference using the pooled baseline standard deviation and applying the small-sample bias correction.*

Bib, bibliotherapy intervention; CI, confidence interval; DSI, digital self-help intervention; GAD-7, Generalized Anxiety Disorder-7; GARS-J, Japanese version of Gaze Anxiety Rating Scale; LSAS, Liebowitz Social Anxiety Scale; MD, mean difference; PHQ-9, Patient Health Questionnaire-9; SPIN, Social Phobia Inventory; WLC, waitlist control; 3M, 3-month follow-up; 6M, 6-month follow-up.

# **Appendix 11: Results of sensitivity analyses for missing not at random**

## Table S5. Sensitivity analysis assuming poorer outcomes among participants with missing data in the Bib group only

| Outcome / time | Comparison | MAR (δ = 0) | δ = 0.20 | δ = 0.50 | δ = 0.80 |
| --- | --- | --- | --- | --- | --- |
| GARS-J / Post | Bib | −10.09 (−14.58, −5.59) | −9.85 (−14.35, −5.34) | −9.48 (−14.02, −4.95) | −9.12 (−13.70, −4.54) |
|  | DSI | −6.95 (−12.07, −1.84) | −6.85 (−11.98, −1.73) | −6.70 (−11.86, −1.55) | −6.55 (−11.75, −1.35) |
| GARS-J / 3M | Bib | −8.02 (−12.61, −3.43) | −7.49 (−12.08, −2.89) | −6.68 (−11.31, −2.06) | −5.88 (−10.55, −1.21) |
|  | DSI | −6.49 (−11.67, −1.31) | −6.39 (−11.58, −1.20) | −6.24 (−11.46, −1.02) | −6.09 (−11.35, −0.82) |
| GARS-J / 6M | Bib | −8.80 (−13.42, −4.17) | −8.21 (−12.85, −3.57) | −7.33 (−12.00, −2.67) | −6.45 (−11.16, −1.74) |
|  | DSI | −8.39 (−13.74, −3.04) | −8.29 (−13.65, −2.93) | −8.14 (−13.53, −2.75) | −7.99 (−13.42, −2.56) |
| LSAS / Post | Bib | −13.57 (−20.81, −6.34) | −13.21 (−20.47, −5.95) | −12.67 (−19.99, −5.36) | −12.13 (−19.54, −4.73) |
|  | DSI | −12.47 (−20.71, −4.24) | −12.32 (−20.57, −4.06) | −12.08 (−20.40, −3.77) | −11.85 (−20.25, −3.45) |
| LSAS / 3M | Bib | −13.64 (−21.11, −6.17) | −12.74 (−20.23, −5.25) | −11.38 (−18.93, −3.84) | −10.03 (−17.66, −2.40) |
|  | DSI | −9.67 (−18.18, −1.16) | −9.51 (−18.04, −0.98) | −9.28 (−17.86, −0.69) | −9.04 (−17.71, −0.37) |
| LSAS / 6M | Bib | −10.50 (−18.14, −2.85) | −9.49 (−17.16, −1.82) | −7.98 (−15.70, −0.26) | −6.47 (−14.28, 1.34) |
|  | DSI | −11.77 (−20.42, −3.11) | −11.61 (−20.29, −2.93) | −11.37 (−20.11, −2.64) | −11.14 (−19.95, −2.33) |
| SPIN / Post | Bib | −6.25 (−10.39, −2.10) | −6.03 (−10.19, −1.88) | −5.71 (−9.90, −1.53) | −5.40 (−9.62, −1.17) |
|  | DSI | −7.42 (−12.08, −2.76) | −7.33 (−12.00, −2.66) | −7.20 (−11.90, −2.49) | −7.07 (−11.81, −2.32) |
| SPIN / 3M | Bib | −4.66 (−8.91, −0.40) | −4.15 (−8.41, 0.11) | −3.39 (−7.68, 0.91) | −2.63 (−6.96, 1.70) |
|  | DSI | −4.70 (−9.48, 0.08) | −4.61 (−9.40, 0.18) | −4.48 (−9.30, 0.34) | −4.35 (−9.21, 0.51) |
| SPIN / 6M | Bib | −5.87 (−10.25, −1.49) | −5.32 (−9.71, −0.93) | −4.51 (−8.92, −0.09) | −3.71 (−8.15, 0.74) |
|  | DSI | −5.63 (−10.38, −0.87) | −5.54 (−10.30, −0.77) | −5.40 (−10.20, −0.61) | −5.27 (−10.11, −0.43) |
| PHQ-9 / Post | Bib | −1.70 (−3.21, −0.19) | −1.62 (−3.14, −0.11) | −1.51 (−3.03, 0.01) | −1.39 (−2.93, 0.15) |
|  | DSI | −2.61 (−4.21, −1.00) | −2.58 (−4.19, −0.97) | −2.54 (−4.16, −0.92) | −2.49 (−4.13, −0.86) |
|  |  |  |  |  | Continued on next pages. |
|  |  |  |  |  |  |
| Table S5 (continued) |  |  |  |  |  |
| Outcome / time | Comparison | MAR (δ = 0) | δ = 0.20 | δ = 0.50 | δ = 0.80 |
| PHQ-9 / 3M | Bib | −0.38 (−2.01, 1.25) | −0.21 (−1.84, 1.42) | 0.05 (−1.59, 1.69) | 0.31 (−1.35, 1.96) |
|  | DSI | −1.04 (−2.73, 0.64) | −1.01 (−2.70, 0.68) | −0.97 (−2.67, 0.73) | −0.93 (−2.64, 0.79) |
| PHQ-9 / 6M | Bib | −0.97 (−2.53, 0.58) | −0.77 (−2.33, 0.79) | −0.46 (−2.03, 1.11) | −0.15 (−1.74, 1.43) |
|  | DSI | −2.34 (−4.04, −0.64) | −2.32 (−4.02, −0.61) | −2.27 (−3.98, −0.56) | −2.23 (−3.96, −0.50) |
| GAD-7 / Post | Bib | −0.99 (−2.23, 0.25) | −0.92 (−2.16, 0.32) | −0.82 (−2.07, 0.42) | −0.72 (−1.98, 0.53) |
|  | DSI | −1.53 (−2.87, −0.19) | −1.51 (−2.85, −0.17) | −1.47 (−2.82, −0.13) | −1.44 (−2.79, −0.08) |
| GAD-7 / 3M | Bib | −0.28 (−1.60, 1.04) | −0.14 (−1.46, 1.17) | 0.06 (−1.26, 1.38) | 0.26 (−1.07, 1.59) |
|  | DSI | −0.19 (−1.61, 1.23) | −0.17 (−1.59, 1.25) | −0.13 (−1.56, 1.29) | −0.10 (−1.53, 1.34) |
| GAD-7 / 6M | Bib | −0.45 (−1.74, 0.84) | −0.28 (−1.57, 1.01) | −0.02 (−1.32, 1.27) | 0.23 (−1.07, 1.54) |
|  | DSI | −0.35 (−1.78, 1.07) | −0.33 (−1.75, 1.10) | −0.29 (−1.72, 1.14) | −0.25 (−1.69, 1.19) |

*Note. Only originally missing follow−up outcomes were modified. The assumed departures from MAR were δ = 0.20, 0.50, and 0.80 times the outcome− and assessment−specific residual standard deviation. Negative adjusted mean differences favour the intervention.*

Bib, bibliotherapy intervention; CI, confidence interval; DSI, digital self−help intervention; GAD-7, Generalized Anxiety Disorder-7; GARS-J, Japanese version of Gaze Anxiety Rating Scale; LSAS, Liebowitz Social Anxiety Scale; MD, mean difference; PHQ-9, Patient Health Questionnaire-9; SPIN, Social Phobia Inventory; WLC, waitlist control; 3M, 3-month follow-up; 6M, 6-month follow-up.

## Table S6. Sensitivity analysis assuming poorer outcomes among participants with missing data in the DSI group only

| Outcome / time | Comparison | MAR (δ = 0) | δ = 0.20 | δ = 0.50 | δ = 0.80 |
| --- | --- | --- | --- | --- | --- |
| GARS-J / Post | Bib | −10.09 (−14.58, −5.59) | −10.08 (−14.58, −5.57) | −10.06 (−14.59, −5.52) | −10.04 (−14.63, −5.44) |
|  | DSI | −6.95 (−12.07, −1.84) | −6.25 (−11.37, −1.12) | −5.19 (−10.34, −0.03) | −4.13 (−9.34, 1.09) |
| GARS-J / 3M | Bib | −8.02 (−12.61, −3.43) | −8.01 (−12.60, −3.41) | −7.99 (−12.61, −3.36) | −7.97 (−12.65, −3.29) |
|  | DSI | −6.49 (−11.67, −1.31) | −5.67 (−10.85, −0.48) | −4.44 (−9.66, 0.78) | −3.21 (−8.49, 2.07) |
| GARS-J / 6M | Bib | −8.80 (−13.42, −4.17) | −8.78 (−13.42, −4.15) | −8.77 (−13.43, −4.10) | −8.75 (−13.47, −4.02) |
|  | DSI | −8.39 (−13.74, −3.04) | −7.56 (−12.92, −2.20) | −6.32 (−11.71, −0.93) | −5.08 (−10.53, 0.37) |
|  |  |  |  |  | Continued on next pages. |
| Table S6 (continued) |  |  |  |  |  |
| Outcome / time | Comparison | MAR (δ = 0) | δ = 0.20 | δ = 0.50 | δ = 0.80 |
| LSAS / Post | Bib | −13.57 (−20.81, −6.34) | −13.55 (−20.80, −6.31) | −13.53 (−20.82, −6.24) | −13.50 (−20.88, −6.12) |
|  | DSI | −12.47 (−20.71, −4.24) | −11.46 (−19.71, −3.22) | −9.95 (−18.24, −1.66) | −8.43 (−16.81, −0.05) |
| LSAS / 3M | Bib | −13.64 (−21.11, −6.17) | −13.62 (−21.10, −6.15) | −13.60 (−21.12, −6.07) | −13.57 (−21.18, −5.96) |
|  | DSI | −9.67 (−18.18, −1.16) | −8.28 (−16.80, 0.23) | −6.21 (−14.77, 2.35) | −4.13 (−12.78, 4.52) |
| LSAS / 6M | Bib | −10.50 (−18.14, −2.85) | −10.48 (−18.13, −2.82) | −10.45 (−18.15, −2.75) | −10.42 (−18.21, −2.64) |
|  | DSI | −11.77 (−20.42, −3.11) | −10.35 (−19.01, −1.68) | −8.21 (−16.92, 0.49) | −6.08 (−14.88, 2.71) |
| SPIN / Post | Bib | −6.25 (−10.39, −2.10) | −6.24 (−10.39, −2.08) | −6.22 (−10.40, −2.04) | −6.20 (−10.43, −1.97) |
|  | DSI | −7.42 (−12.08, −2.76) | −6.82 (−11.49, −2.15) | −5.93 (−10.62, −1.23) | −5.04 (−9.78, −0.30) |
| SPIN / 3M | Bib | −4.66 (−8.91, −0.40) | −4.65 (−8.91, −0.39) | −4.63 (−8.92, −0.34) | −4.61 (−8.95, −0.28) |
|  | DSI | −4.70 (−9.48, 0.08) | −3.93 (−8.71, 0.86) | −2.76 (−7.58, 2.06) | −1.60 (−6.46, 3.26) |
| SPIN / 6M | Bib | −5.87 (−10.25, −1.49) | −5.86 (−10.25, −1.47) | −5.84 (−10.26, −1.42) | −5.82 (−10.29, −1.36) |
|  | DSI | −5.63 (−10.38, −0.87) | −4.86 (−9.62, −0.10) | −3.71 (−8.50, 1.07) | −2.59 (−7.42, 2.24) |
| PHQ-9 / Post | Bib | −1.70 (−3.21, −0.19) | −1.70 (−3.21, −0.19) | −1.69 (−3.21, −0.17) | −1.68 (−3.22, −0.14) |
|  | DSI | −2.61 (−4.21, −1.00) | −2.39 (−4.00, −0.78) | −2.07 (−3.69, −0.45) | −1.74 (−3.38, −0.10) |
| PHQ-9 / 3M | Bib | −0.38 (−2.01, 1.25) | −0.38 (−2.01, 1.26) | −0.37 (−2.01, 1.27) | −0.36 (−2.02, 1.30) |
|  | DSI | −1.04 (−2.73, 0.64) | −0.78 (−2.47, 0.91) | −0.39 (−2.09, 1.31) | 0.00 (−1.72, 1.72) |
| PHQ-9 / 6M | Bib | −0.97 (−2.53, 0.58) | −0.97 (−2.52, 0.59) | −0.96 (−2.53, 0.61) | −0.95 (−2.54, 0.63) |
|  | DSI | −2.34 (−4.04, −0.64) | −2.06 (−3.76, −0.36) | −1.63 (−3.34, 0.09) | −1.20 (−2.93, 0.53) |
| GAD-7 / Post | Bib | −0.99 (−2.23, 0.25) | −0.99 (−2.23, 0.25) | −0.98 (−2.23, 0.27) | −0.98 (−2.24, 0.29) |
|  | DSI | −1.53 (−2.87, −0.19) | −1.35 (−2.69, −0.01) | −1.08 (−2.43, 0.27) | −0.81 (−2.18, 0.56) |
| GAD-7 / 3M | Bib | −0.28 (−1.60, 1.04) | −0.28 (−1.59, 1.04) | −0.27 (−1.60, 1.05) | −0.27 (−1.61, 1.07) |
|  | DSI | −0.19 (−1.61, 1.23) | 0.01 (−1.41, 1.43) | 0.31 (−1.12, 1.75) | 0.62 (−0.83, 2.07) |
| GAD-7 / 6M | Bib | −0.45 (−1.74, 0.84) | −0.45 (−1.74, 0.84) | −0.44 (−1.74, 0.86) | −0.44 (−1.75, 0.87) |
|  | DSI | −0.35 (−1.78, 1.07) | −0.11 (−1.54, 1.32) | 0.25 (−1.19, 1.68) | 0.60 (−0.85, 2.06) |

*Note. Only originally missing follow−up outcomes were modified. The assumed departures from MAR were δ = 0.20, 0.50, and 0.80 times the outcome− and assessment−specific residual standard deviation. Negative adjusted mean differences favour the intervention.*

Bib, bibliotherapy intervention; CI, confidence interval; DSI, digital self−help intervention; GAD-7, Generalized Anxiety Disorder-7; GARS-J, Japanese version of Gaze Anxiety Rating Scale; LSAS, Liebowitz Social Anxiety Scale; MD, mean difference; PHQ-9, Patient Health Questionnaire-9; SPIN, Social Phobia Inventory; WLC, waitlist control; 3M, 3-month follow-up; 6M, 6-month follow-up.

## Table S7. Sensitivity analysis assuming better outcomes among participants with missing data in the waitlist control group only

| Outcome / time | Comparison | MAR (δ = 0) | δ = 0.20 | δ = 0.50 | δ = 0.80 |
| --- | --- | --- | --- | --- | --- |
| GARS-J / Post | Bib | −10.09 (−14.58, −5.59) | −9.91 (−14.41, −5.40) | −9.63 (−14.16, −5.11) | −9.36 (−13.92, −4.80) |
|  | DSI | −6.95 (−12.07, −1.84) | −6.80 (−11.92, −1.67) | −6.56 (−11.71, −1.41) | −6.33 (−11.51, −1.15) |
| GARS-J / 3M | Bib | −8.02 (−12.61, −3.43) | −7.73 (−12.32, −3.14) | −7.29 (−11.91, −2.68) | −6.86 (−11.51, −2.21) |
|  | DSI | −6.49 (−11.67, −1.31) | −6.22 (−11.41, −1.03) | −5.82 (−11.03, −0.61) | −5.43 (−10.67, −0.18) |
| GARS-J / 6M | Bib | −8.80 (−13.42, −4.17) | −8.37 (−13.01, −3.74) | −7.74 (−12.40, −3.08) | −7.11 (−11.79, −2.42) |
|  | DSI | −8.39 (−13.74, −3.04) | −7.99 (−13.35, −2.63) | −7.40 (−12.78, −2.02) | −6.80 (−12.21, −1.39) |
| LSAS / Post | Bib | −13.57 (−20.81, −6.34) | −13.30 (−20.55, −6.05) | −12.89 (−20.18, −5.61) | −12.48 (−19.82, −5.15) |
|  | DSI | −12.47 (−20.71, −4.24) | −12.24 (−20.48, −3.99) | −11.88 (−20.17, −3.60) | −11.53 (−19.87, −3.20) |
| LSAS / 3M | Bib | −13.64 (−21.11, −6.17) | −13.15 (−20.63, −5.67) | −12.42 (−19.93, −4.90) | −11.69 (−19.26, −4.12) |
|  | DSI | −9.67 (−18.18, −1.16) | −9.21 (−17.73, −0.69) | −8.54 (−17.09, 0.02) | −7.86 (−16.47, 0.75) |
| LSAS / 6M | Bib | −10.50 (−18.14, −2.85) | −9.77 (−17.43, −2.11) | −8.69 (−16.38, −1.00) | −7.61 (−15.35, 0.12) |
|  | DSI | −11.77 (−20.42, −3.11) | −11.08 (−19.75, −2.41) | −10.05 (−18.75, −1.35) | −9.03 (−17.78, −0.28) |
| SPIN / Post | Bib | −6.25 (−10.39, −2.10) | −6.08 (−10.24, −1.93) | −5.84 (−10.02, −1.66) | −5.60 (−9.81, −1.39) |
|  | DSI | −7.42 (−12.08, −2.76) | −7.28 (−11.96, −2.61) | −7.08 (−11.77, −2.38) | −6.87 (−11.60, −2.14) |
| SPIN / 3M | Bib | −4.66 (−8.91, −0.40) | −4.38 (−8.65, −0.12) | −3.98 (−8.26, 0.30) | −3.59 (−7.90, 0.72) |
|  | DSI | −4.70 (−9.48, 0.08) | −4.45 (−9.24, 0.34) | −4.08 (−8.90, 0.73) | −3.73 (−8.58, 1.12) |
| SPIN / 6M | Bib | −5.87 (−10.25, −1.49) | −5.45 (−9.84, −1.05) | −4.82 (−9.23, −0.41) | −4.21 (−8.65, 0.22) |
|  | DSI | −5.63 (−10.38, −0.87) | −5.23 (−9.99, −0.46) | −4.64 (−9.43, 0.15) | −4.06 (−8.88, 0.75) |
|  |  |  |  |  | Continued on next pages. |
|  |  |  |  |  |  |
| Table S7 (continued) |  |  |  |  |  |
| Outcome / time | Comparison | MAR (δ = 0) | δ = 0.20 | δ = 0.50 | δ = 0.80 |
| PHQ-9 / Post | Bib | −1.70 (−3.21, −0.19) | −1.65 (−3.16, −0.13) | −1.56 (−3.08, −0.05) | −1.49 (−3.01, 0.04) |
|  | DSI | −2.61 (−4.21, −1.00) | −2.56 (−4.17, −0.95) | −2.49 (−4.11, −0.87) | −2.42 (−4.05, −0.80) |
| PHQ-9 / 3M | Bib | −0.38 (−2.01, 1.25) | −0.29 (−1.93, 1.34) | −0.17 (−1.81, 1.46) | −0.07 (−1.71, 1.56) |
|  | DSI | −1.04 (−2.73, 0.64) | −0.96 (−2.65, 0.72) | −0.86 (−2.55, 0.84) | −0.76 (−2.47, 0.94) |
| PHQ-9 / 6M | Bib | −0.97 (−2.53, 0.58) | −0.83 (−2.39, 0.73) | −0.63 (−2.19, 0.94) | −0.45 (−2.01, 1.12) |
|  | DSI | −2.34 (−4.04, −0.64) | −2.21 (−3.91, −0.50) | −2.02 (−3.73, −0.31) | −1.85 (−3.56, −0.14) |
| GAD-7 / Post | Bib | −0.99 (−2.23, 0.25) | −0.94 (−2.18, 0.30) | −0.88 (−2.12, 0.36) | −0.82 (−2.06, 0.41) |
|  | DSI | −1.53 (−2.87, −0.19) | −1.49 (−2.83, −0.15) | −1.44 (−2.78, −0.10) | −1.39 (−2.73, −0.05) |
| GAD-7 / 3M | Bib | −0.28 (−1.60, 1.04) | −0.23 (−1.54, 1.09) | −0.16 (−1.47, 1.15) | −0.10 (−1.41, 1.20) |
|  | DSI | −0.19 (−1.61, 1.23) | −0.15 (−1.57, 1.27) | −0.09 (−1.50, 1.33) | −0.04 (−1.45, 1.38) |
| GAD-7 / 6M | Bib | −0.45 (−1.74, 0.84) | −0.36 (−1.64, 0.93) | −0.24 (−1.52, 1.04) | −0.16 (−1.43, 1.11) |
|  | DSI | −0.35 (−1.78, 1.07) | −0.26 (−1.69, 1.16) | −0.16 (−1.57, 1.25) | −0.08 (−1.48, 1.32) |

*Note. Only originally missing follow−up outcomes were modified. The assumed departures from MAR were δ = 0.20, 0.50, and 0.80 times the outcome− and assessment−specific residual standard deviation. Negative adjusted mean differences favour the intervention.*

Bib, bibliotherapy intervention; CI, confidence interval; DSI, digital self−help intervention; GAD-7, Generalized Anxiety Disorder-7; GARS-J, Japanese version of Gaze Anxiety Rating Scale; LSAS, Liebowitz Social Anxiety Scale; MD, mean difference; PHQ-9, Patient Health Questionnaire-9; SPIN, Social Phobia Inventory; WLC, waitlist control; 3M, 3-month follow-up; 6M, 6-month follow-up.

## Table S8. Sensitivity analysis assuming poorer outcomes among participants with missing data in both intervention groups

| Outcome / time | Comparison | MAR (δ = 0) | δ = 0.20 | δ = 0.50 | δ = 0.80 |
| --- | --- | --- | --- | --- | --- |
| GARS-J / Post | Bib | −10.09 (−14.58, −5.59) | −9.83 (−14.35, −5.32) | −9.45 (−14.02, −4.88) | −9.07 (−13.74, −4.39) |
|  | DSI | −6.95 (−12.07, −1.84) | −6.15 (−11.28, −1.01) | −4.94 (−10.13, 0.26) | −3.73 (−9.02, 1.57) |
| GARS-J / 3M | Bib | −8.02 (−12.61, −3.43) | −7.47 (−12.08, −2.87) | −6.65 (−11.31, −1.99) | −5.83 (−10.59, −1.07) |
|  | DSI | −6.49 (−11.67, −1.31) | −5.57 (−10.76, −0.37) | −4.19 (−9.45, 1.07) | −2.81 (−8.17, 2.55)  Continued on next pages. |
| Table S8 (continued) |  |  |  |  |  |
| Outcome / time | Comparison | MAR (δ = 0) | δ = 0.20 | δ = 0.50 | δ = 0.80 |
| GARS-J / 6M | Bib | −8.80 (−13.42, −4.17) | −8.20 (−12.84, −5.55) | −7.30 (−12.00, −2.60) | −6.40 (−11.20, −1.60) |
|  | DSI | −8.39 (−13.74, −3.04) | −7.46 (−12.83, −2.09) | −6.07 (−11.50, −0.65) | −4.68 (−10.20, 0.84) |
| LSAS / Post | Bib | −13.57 (−20.81, −6.34) | −13.19 (−20.46, −5.93) | −12.63 (−20.00, −5.26) | −12.06 (−19.60, −4.52) |
|  | DSI | −12.47 (−20.71, −4.24) | −11.31 (−19.57, −3.04) | −9.56 (−17.92, −1.19) | −7.81 (−16.34, 0.73) |
| LSAS / 3M | Bib | −13.64 (−21.11, −6.17) | −12.72 (−20.22, −5.22) | −11.34 (−18.94, −3.74) | −9.96 (−17.72, −2.20) |
|  | DSI | −9.67 (−18.18, −1.16) | −8.13 (−16.66, 0.41) | −5.81 (−14.45, 2.82) | −3.50 (−12.30, 5.30) |
| LSAS / 6M | Bib | −10.50 (−18.14, −2.85) | −9.47 (−17.15, −1.80) | −7.93 (−15.71, −0.16) | −6.40 (−14.33, 1.54) |
|  | DSI | −11.77 (−20.42, −3.11) | −10.19 (−18.87, −1.51) | −7.82 (−16.60, 0.96) | −5.46 (−14.40, 3.49) |
| SPIN / Post | Bib | −6.25 (−10.39, −2.10) | −6.02 (−10.18, −1.86) | −5.68 (−9.90, −1.46) | −5.35 (−9.65, −1.05) |
|  | DSI | −7.42 (−12.08, −2.76) | −6.73 (−11.41, −2.05) | −5.70 (−10.43, −0.97) | −4.68 (−9.50, 0.13) |
| SPIN / 3M | Bib | −4.66 (−8.91, −0.40) | −4.14 (−8.41, 0.13) | −3.36 (−7.68, 0.97) | −2.58 (−6.99, 1.82) |
|  | DSI | −4.70 (−9.48, 0.08) | −3.84 (−8.63, 0.96) | −2.54 (−7.39, 2.32) | −1.25 (−6.18, 3.68) |
| SPIN / 6M | Bib | −5.87 (−10.25, −1.49) | −5.31 (−9.71, −0.91) | −4.48 (−8.92, −0.03) | −3.66 (−8.18, 0.86) |
|  | DSI | −5.63 (−10.38, −0.87) | −4.77 (−9.54, 0.00) | −3.49 (−8.31, 1.33) | −2.24 (−7.14, 2.67) |
| PHQ-9 / Post | Bib | −1.70 (−3.21, −0.19) | −1.62 (−3.13, −0.11) | −1.49 (−3.03, 0.04) | −1.37 (−2.94, 0.20) |
|  | DSI | −2.61 (−4.21, −1.00) | −2.36 (−3.97, −0.75) | −1.99 (−3.63, −0.36) | −1.63 (−3.29, 0.04) |
| PHQ-9 / 3M | Bib | −0.38 (−2.01, 1.25) | −0.20 (−1.84, 1.43) | 0.06 (−1.59, 1.71) | 0.33 (−1.36, 2.01) |
|  | DSI | −1.04 (−2.73, 0.64) | −0.75 (−2.44, 0.94) | −0.32 (−2.03, 1.39) | 0.12 (−1.63, 1.86) |
| PHQ-9 / 6M | Bib | −0.97 (−2.53, 0.58) | −0.76 (−2.32, 0.80) | −0.45 (−2.03, 1.13) | −0.13 (−1.75, 1.48) |
|  | DSI | −2.34 (−4.04, −0.64) | −2.03 (−3.73, −0.32) | −1.56 (−3.28, 0.17) | −1.08 (−2.84, 0.67) |
| GAD-7 / Post | Bib | −0.99 (−2.23, 0.25) | −0.92 (−2.16, 0.32) | −0.82 (−2.07, 0.44) | −0.71 (−1.99, 0.57) |
|  | DSI | −1.53 (−2.87, −0.19) | −1.33 (−2.67, 0.01) | −1.02 (−2.38, 0.34) | −0.71 (−2.09, 0.67) |
|  |  |  |  |  | Continued on next pages. |
| Table S8 (continued) |  |  |  |  |  |
| Outcome / time | Comparison | MAR (δ = 0) | δ = 0.20 | δ = 0.50 | δ = 0.80 |
| GAD-7 / 3M | Bib | −0.28 (−1.60, 1.04) | −0.14 (−1.46, 1.18) | 0.07 (−1.27, 1.40) | 0.27 (−1.08, 1.63) |
|  | DSI | −0.19 (−1.61, 1.23) | 0.03 (−1.39, 1.46) | 0.38 (−1.06, 1.81) | 0.72 (−0.74, 2.18) |
| GAD-7 / 6M | Bib | −0.45 (−1.74, 0.84) | −0.28 (−1.57, 1.02) | −0.01 (−1.32, 1.29) | 0.25 (−1.08, 1.57) |
|  | DSI | −0.35 (−1.78, 1.07) | −0.09 (−1.52, 1.34) | 0.31 (−1.13, 1.75) | 0.70 (−0.76, 2.17) |

*Note. Only originally missing follow−up outcomes were modified. The assumed departures from MAR were δ = 0.20, 0.50, and 0.80 times the outcome− and assessment−specific residual standard deviation. Negative adjusted mean differences favour the intervention.*

Bib, bibliotherapy intervention; CI, confidence interval; DSI, digital self−help intervention; GAD-7, Generalized Anxiety Disorder-7; GARS-J, Japanese version of Gaze Anxiety Rating Scale; LSAS, Liebowitz Social Anxiety Scale; MD, mean difference; PHQ-9, Patient Health Questionnaire-9; SPIN, Social Phobia Inventory; WLC, waitlist control; 3M, 3-month follow-up; 6M, 6-month follow-up.

# **Appendix 12: Details of dichotomous outcomes**

## Table S9. Pooled proportions and risk ratios for clinically significant outcomes relative to the waitlist control

| Outcome | Group | Post Pooled % | RR (95% CI) | 3M Pooled % | RR (95% CI) | 6M Pooled % | RR (95% CI) |
| --- | --- | --- | --- | --- | --- | --- | --- |
| Response |  |  |  |  |  |  |  |
|  | Bib | 34.6 | 5.51 (2.06, 14.72) | 41.0 | 1.86 (1.10, 3.14) | 40.7 | 3.02 (1.56, 5.82) |
|  | DSI | 28.8 | 4.57 (1.65, 12.65) | 36.4 | 1.65 (0.93, 2.91) | 37.7 | 2.78 (1.40, 5.56) |
|  | WLC | 6.4 | Reference | 22.1 | Reference | 13.6 | Reference |
| Remission |  |  |  |  |  |  |  |
|  | Bib | 21.1 | 2.88 (1.13, 7.36) | 33.2 | 2.17 (1.14, 4.13) | 26.9 | 2.85 (1.24, 6.56) |
|  | DSI | 24.3 | 3.31 (1.31, 8.37) | 26.7 | 1.73 (0.85, 3.51) | 27.3 | 2.88 (1.22, 6.81) |
|  | WLC | 7.4 | Reference | 15.4 | Reference | 9.5 | Reference |
| Recovery |  |  |  |  |  |  |  |
|  | Bib | 26.0 | 1.85 (0.93, 3.66) | 36.0 | 1.91 (1.07, 3.41) | 34.6 | 1.98 (1.09, 3.60) |
|  | DSI | 31.9 | 2.26 (1.16, 4.42) | 32.8 | 1.74 (0.94, 3.21) | 36.9 | 2.11 (1.15, 3.86) |
|  | WLC | 14.1 | Reference | 18.9 | Reference | 17.5 | Reference |
| Deterioration |  |  |  |  |  |  |  |
|  | Bib | 9.1 | 0.94 (0.34, 2.60) | 7.9 | 0.42 (0.16, 1.08) | 13.0 | 0.95 (0.39, 2.32) |
|  | DSI | 6.0 | 0.60 (0.16, 2.20) | 9.4 | 0.49 (0.19, 1.28) | 9.8 | 0.71 (0.25, 2.03) |
|  | WLC | 9.7 | Reference | 18.8 | Reference | 13.7 | Reference |

*Note. Percentages are pooled estimates across 50 multiply imputed datasets; therefore, a single integer event count is not reported. Risk ratios and their 95% confidence intervals were estimated using modified Poisson regression with robust standard errors and combined across imputations using Rubin’s rules. Response was defined as a ≥28% decrease from baseline in the Liebowitz Social Anxiety Scale (LSAS) total score; remission as an LSAS total score <35; recovery as both a Social Phobia Inventory total score <19 and a Patient Health Questionnaire-9 total score <10; and deterioration as a ≥28% increase in the LSAS total score.*

Bib, bibliotherapy intervention; CI, confidence interval; DSI, digital self-help intervention; RR, risk ratio; WLC, waitlist control; 3M, 3-month follow-up; 6M, 6-month follow-up.

## Table S10. Pooled proportions and odds ratios for clinically significant outcomes relative to the waitlist control

| Outcome | Group | Post Pooled % | OR (95% CI) | 3M Pooled % | OR (95% CI) | 6M Pooled % | OR (95% CI) |
| --- | --- | --- | --- | --- | --- | --- | --- |
| Response |  |  |  |  |  |  |  |
|  | Bib | 34.6 | 7.89 (2.62, 23.80) | 41.0 | 2.46 (1.17, 5.16) | 40.7 | 4.40 (1.92, 10.10) |
|  | DSI | 28.8 | 6.02 (1.91, 18.99) | 36.4 | 2.02 (0.91, 4.50) | 37.7 | 3.87 (1.60, 9.37) |
|  | WLC | 6.4 | Reference | 22.1 | Reference | 13.6 | Reference |
| Remission |  |  |  |  |  |  |  |
|  | Bib | 21.1 | 3.38 (1.19, 9.67) | 33.2 | 2.75 (1.22, 6.22) | 26.9 | 3.53 (1.33, 9.33) |
|  | DSI | 24.3 | 4.05 (1.42, 11.58) | 26.7 | 2.00 (0.82, 4.88) | 27.3 | 3.59 (1.30, 9.87) |
|  | WLC | 7.4 | Reference | 15.4 | Reference | 9.5 | Reference |
| Recovery |  |  |  |  |  |  |  |
|  | Bib | 26.0 | 2.14 (0.93, 4.94) | 36.0 | 2.42 (1.12, 5.23) | 34.6 | 2.49 (1.14, 5.46) |
|  | DSI | 31.9 | 2.86 (1.24, 6.61) | 32.8 | 2.10 (0.93, 4.74) | 36.9 | 2.76 (1.24, 6.15) |
|  | WLC | 14.1 | Reference | 18.9 | Reference | 17.5 | Reference |
| Deterioration |  |  |  |  |  |  |  |
|  | Bib | 9.1 | 0.94 (0.31, 2.87) | 7.9 | 0.37 (0.13, 1.07) | 13.0 | 0.94 (0.34, 2.63) |
|  | DSI | 6.0 | 0.57 (0.14, 2.33) | 9.4 | 0.44 (0.15, 1.30) | 9.8 | 0.68 (0.21, 2.21) |
|  | WLC | 9.7 | Reference | 18.8 | Reference | 13.7 | Reference |

*Note. Percentages are pooled estimates across 50 multiply imputed datasets; therefore, a single integer event count is not reported. Odds ratios and their 95% confidence intervals were estimated using logistic regression and combined across imputations using Rubin’s rules. Response was defined as a ≥28% decrease from baseline in the Liebowitz Social Anxiety Scale (LSAS) total score; remission as an LSAS total score <35; recovery as both a Social Phobia Inventory total score <19 and a Patient Health Questionnaire-9 total score <10; and deterioration as a ≥28% increase in the LSAS total score.*

Bib, bibliotherapy intervention; CI, confidence interval; DSI, digital self-help intervention; OR, odds ratio; WLC, waitlist control; 3M, 3-month follow-up; 6M, 6-month follow-up.

## Figure S1. Sensitivity of adjusted treatment effects on gaze-related anxiety to alternative assumptions about missing data

*Note. Adjusted mean differences in GARS-J between each intervention group and the waitlist control group are presented at post-intervention, 3-month follow-up, and 6-month follow-up. The four lines represent different missing-data scenarios: poorer outcomes in the bibliotherapy (Bib) group only, the digital self-help (DSI) group only, or both intervention groups; and better outcomes in the waitlist control group only. The horizontal axis indicates the degree of departure from the missing-at-random assumption (δ = 0, 0.20, 0.50, and 0.80), expressed in residual standard deviation units. Negative values indicate outcomes favouring the intervention. Across all scenarios and assessment points, adjusted mean differences remained negative. Estimates for Bib were relatively stable across scenarios. The estimated treatment effects for the DSI showed greater attenuation under scenarios assuming poorer outcomes among participants with missing data in the DSI group, either alone or in both intervention groups.*

Bib, bibliotherapy; DSI, digital self-help intervention; SD, standard deviation; CI, confidence interval; GARS-J, Gaze Anxiety Rating Scale–Japanese version.

## Figure S2. Sensitivity of adjusted treatment effects on the Liebowitz Social Anxiety Scale to alternative assumptions about missing data

*Note. Adjusted mean differences in the Liebowitz Social Anxiety Scale scores between each intervention group and the waitlist control group are presented at post-intervention, 3-month follow-up, and 6-month follow-up. Negative values indicate outcomes favouring the intervention. Across all scenarios and assessment points, adjusted mean differences remained negative. The estimated treatment effects for Bib were relatively stable across scenarios. The estimated treatment effects for DSI showed particularly pronounced attenuation under scenarios assuming poorer outcomes among participants with missing data in the DSI group, either alone or in both intervention groups.*

Bib, bibliotherapy; DSI, digital self-help intervention; SD, standard deviation; CI, confidence interval.

Figure S3. Sensitivity of adjusted treatment effects on the Social Phobia Inventory to alternative assumptions about missing data

*Note. Adjusted mean differences in the Generalized Anxiety Disorder-7 scores between each intervention group and the waitlist control group are presented at post-intervention, 3-month follow-up, and 6-month follow-up. Negative values indicate outcomes favouring the intervention. At post-intervention, adjusted mean differences remained negative across all scenarios. However, at 3-month and 6-month follow-up, some estimates approached or crossed zero under increasingly unfavourable assumptions about missing outcomes. The estimated treatment effects for Bib showed pronounced attenuation under scenarios assuming poorer outcomes among participants with missing data in the Bib group, whereas those for DSI showed particularly pronounced attenuation under scenarios assuming poorer outcomes among participants with missing data in the DSI group, either alone or in both intervention groups.*

Bib, bibliotherapy; DSI, digital self-help intervention; SD, standard deviation; CI, confidence interval.

Figure S4. Sensitivity of adjusted treatment effects on the Patient Health Questionnaire-9 to alternative assumptions about missing data

*Note. Adjusted mean differences in the PHQ-9 scores between each intervention group and the waitlist control group are presented at post-intervention, 3-month follow-up, and 6-month follow-up. Negative values indicate outcomes favouring the intervention. Adjusted mean differences remained negative across all scenarios at post-intervention and 6-month follow-up. However, at 3-month follow-up, some estimates approached or crossed zero under increasingly unfavourable assumptions about missing outcomes. The estimated treatment effects for Bib showed attenuation under scenarios assuming poorer outcomes among participants with missing data in the Bib group, whereas those for DSI showed particularly pronounced attenuation under scenarios assuming poorer outcomes among participants with missing data in the DSI group, either alone or in both intervention groups.*

Bib, bibliotherapy; DSI, digital self-help intervention; SD, standard deviation; CI, confidence interval; PHQ-9, Patient Health Questionnaire-9

Figure S5. Sensitivity of adjusted treatment effects on the Generalized Anxiety Disorder-7 to alternative assumptions about missing data

*Note. Adjusted mean differences in the Generalized Anxiety Disorder-7 scores between each intervention group and the waitlist control group are presented at post-intervention, 3-month follow-up, and 6-month follow-up. Negative values indicate outcomes favouring the intervention. At post-intervention, adjusted mean differences remained negative across all scenarios. However, at 3-month and 6-month follow-up, some estimates approached or crossed zero under increasingly unfavourable assumptions about missing outcomes. The estimated treatment effects for Bib showed pronounced attenuation under scenarios assuming poorer outcomes among participants with missing data in the Bib group, whereas those for DSI showed particularly pronounced attenuation under scenarios assuming poorer outcomes among participants with missing data in the DSI group, either alone or in both intervention groups.*

Bib, bibliotherapy; DSI, digital self-help intervention; SD, standard deviation; CI, confidence interval; GAD-7, Generalized Anxiety Disorder-7.

# **Appendix 13: All adverse events each group**

## Table S11. Adverse event and serious adverse events during this trial

|  | **Waitlist control** | **Bib** | **DSI** |
| --- | --- | --- | --- |
| **Adverse Events** |  |  |  |
| Depression | 3 | 4 | 2 |
| Mania | 1 | ― | ― |
| Suicidal ideation | 2 | ― | ― |
| Agitation | 1 | ― | ― |
| Insomnia | 1 | 1 | ― |
| Faver | 5 | 5 | 1 |
| Headache | 2 | 4 |  |
| Common cold (URTI) | 4 | 3 | 2 |
| Abdominal pain | ― | 1 | ― |
| Dysgeusia | ― | 1 | ― |
| Hairline fracture | ― | 1 | ― |
| Sprain | 1 | ― | ― |
| **Serious Adverse Events** |  | ― | ― |
| Suicidal attempt | 1 | ― | ― |

Bib, bibliotherapy intervention; DSI, digital self-help intervention; URTI, upper respiratory tract infection.

# **Appendix 14: Details of absolute risk reduction and number needed to treat analyses**

## Table S12. Results of Deterioration risk analyses

| Time | Comparison | Deterioration, intervention (%) | Deterioration, WLC (%) | ARR, % (95% CI) | NNTB | RR (95% CI) |
| --- | --- | --- | --- | --- | --- | --- |
| Post |  |  |  |  |  |  |
|  | Bib vs WLC | 9.1 | 9.7 | 0.60 (−8.9, 10.0) | 172.8 | 0.94 (0.34, 2.60) |
|  | DSI vs WLC | 6.0 | 9.7 | 3.6 (−5.8, 13.1) | 27.4 | 0.60 (0.16, 2.20) |
| 3M |  |  |  |  |  |  |
|  | Bib vs WLC | 7.9 | 18.8 | 10.9 (−0.2, 21.9) | 9.2 | 0.42 (0.16, 1.08) |
|  | DSI vs WLC | 9.4 | 18.8 | 9.4 (−2.4, 21.2) | 10.6 | 0.49 (0.19, 1.28) |
| 6M |  |  |  |  |  |  |
|  | Bib vs WLC | 13.0 | 13.7 | 0.7 (−11.1, 12.4) | 147.6 | 0.95 (0.39, 2.32) |
|  | DSI vs WLC | 9.8 | 13.7 | 3.9 (−7.9, 15.7) | 25.8 | 0.71 (0.25, 2.03) |

*Note. Deterioration was defined as a ≥28% increase from baseline in the Liebowitz Social Anxiety Scale total score. Percentages are pooled estimates across 50 multiply imputed datasets. Absolute risk reduction (ARR) was calculated as the pooled risk of deterioration in the waitlist control group minus that in the intervention group; positive values therefore indicate a lower risk in the intervention group. The number needed to treat for benefit (NNTB) was calculated as the reciprocal of the unrounded pooled ARR and is presented as a point estimate to aid clinical interpretation. NNTB estimates should be interpreted together with the corresponding 95% CIs for ARR. When the 95% CI for ARR includes zero, the data are compatible with no difference and potentially with effects in either direction; therefore, the NNTB point estimate should not be interpreted as evidence of a preventive effect.*

Bib, bibliotherapy intervention; CI, confidence interval; DSI, digital self-help intervention; NNTB, number needed to treat for benefit; WLC, waitlist control; 3M, 3-month follow-up; 6M, 6-month follow-up.

# **Appendix 15: Complete R code for the primary analyses**

The following R code reproduces the revised descriptive statistics, multiple-imputation procedures, primary mixed-effects analyses, clinically significant outcome analyses, model diagnostics, and corresponding tables and figures. Table 1 and Supplementary Table S2 are generated from the observed pre-imputation data, whereas inferential analyses are based on 50 multiply imputed datasets. The sensitivity analyses used the same analytical workflow, with only the input dataset replaced by the prespecified sensitivity-analysis dataset.

\# ======================================================================

\# Appendix 12. R code for the revised primary analyses

\# ======================================================================

\# The following code reproduces the revised inferential analyses, including multiple imputation, linear mixed-effects models, pooled estimated marginal means and Dunnett-adjusted contrasts, Hedges' g, model diagnostics, clinically significant outcomes, and Figure 2.

\# Table 1 and Supplementary Table S2 are descriptive summaries calculated directly from the observed pre-imputation dataset and are therefore not reproduced by the inferential-analysis code below.

\# The sensitivity analyses used the same analytical workflow, with only the input dataset replaced by the prespecified sensitivity-analysis dataset. Both the primary-analysis and sensitivity-analysis datasets are provided as supplementary data.

\# Assumed allocation coding:

\#	1 = Control / WLC

\#	2 = BookCBT / Bib

\#	3 = WebCBT / DSI

\#

\# Final mixed-effects model:

\#	change from baseline ~ allocation * time + baseline + sex + facility

\#	+ (1 | id)

\# Facility is included as a fixed effect and participant as a random

\# intercept.

\# ======================================================================

\# ------------------------------------------------------------

\# 0. Packages

\# ------------------------------------------------------------

required_packages <- c(

"readxl", "mice", "lme4", "broom.mixed", "emmeans",

"dplyr", "tidyr", "purrr", "writexl", "lattice"

)

to_install <- required_packages[

!vapply(required_packages, requireNamespace, logical(1), quietly = TRUE)

]

if (length(to_install) > 0) {

install.packages(to_install)

}

library(readxl)

library(mice)

library(lme4)

library(broom.mixed)

library(emmeans)

library(dplyr)

library(tidyr)

library(purrr)

library(writexl)

library(lattice)

\# Reproducibility

set.seed(500)

\# Number of imputations and MICE iterations

M <- 50

MAXIT <- 20

\# ------------------------------------------------------------

\# 1. Read master dataset

\# ------------------------------------------------------------

file_path <- "master_dataset.xlsx"

if (!file.exists(file_path)) {

message("master_dataset.xlsx was not found in the working directory.")

message("Please choose the file manually.")

file_path <- file.choose()

}

dat <- read_excel(file_path)

required_cols <- c(

"id", "allocation", "facility", "sex",

"preGARS", "postGARS", "threeGARS", "sixGARS",

"preLSAS", "postLSAS", "threeLSAS", "sixLSAS",

"preSPIN", "postSPIN", "threeSPIN", "sixSPIN",

"prePHQ", "postPHQ", "threePHQ", "sixPHQ",

"preGAD", "postGAD", "threeGAD", "sixGAD"

)

missing_cols <- setdiff(required_cols, names(dat))

if (length(missing_cols) > 0) {

stop(

"The following required columns are missing: ",

paste(missing_cols, collapse = ", ")

)

}

\# Check participant IDs

if (anyDuplicated(dat$id) > 0) {

stop("id is not unique. Check the master dataset before analysis.")

}

\# Factor coding

dat <- dat %>%

mutate(

id = factor(id),

allocation = factor(

allocation,

levels = c(1, 2, 3),

labels = c("Control", "BookCBT", "WebCBT")

),

facility = factor(facility),

sex = factor(sex)

)

\# Variables that must not contain missing values

complete_required <- c(

"id", "allocation", "facility", "sex",

"preGARS", "preLSAS", "preSPIN", "prePHQ", "preGAD"

)

if (anyNA(dat[complete_required])) {

stop(

"Missing values were found in ID/allocation/facility/sex/baseline variables. ",

"Please inspect the dataset before running the primary analysis."

)

}

cat("\nSample size by allocation:\n")

print(table(dat$allocation))

cat("\nSample size by facility and allocation:\n")

print(table(dat$facility, dat$allocation))

cat("\nSample size by sex and allocation:\n")

print(table(dat$sex, dat$allocation))

\# ------------------------------------------------------------

\# 2. Describe missingness before imputation

\# ------------------------------------------------------------

followup_vars <- c(

"postGARS", "threeGARS", "sixGARS",

"postLSAS", "threeLSAS", "sixLSAS",

"postSPIN", "threeSPIN", "sixSPIN",

"postPHQ", "threePHQ", "sixPHQ",

"postGAD", "threeGAD", "sixGAD"

)

missingness_summary <- data.frame(

variable = followup_vars,

n_missing = vapply(dat[followup_vars], function(x) sum(is.na(x)), numeric(1)),

pct_missing = vapply(

dat[followup_vars],

function(x) 100 * mean(is.na(x)),

numeric(1)

)

)

cat("\nMissingness summary:\n")

print(missingness_summary)

cat("\nMissing-data pattern:\n")

md.pattern(dat, plot = FALSE)

\# ------------------------------------------------------------

\# 3. Multiple imputation by chained equations

\# ------------------------------------------------------------

\# Initialise MICE without imputing

ini <- mice(dat, maxit = 0, printFlag = FALSE)

meth <- ini$method

pred <- ini$predictorMatrix

\# ID, allocation, facility, sex are not themselves imputed.

\# Baseline variables are complete and therefore also remain unchanged.

meth[c("id", "allocation", "facility", "sex")] <- ""

\# Do not use participant ID as an imputation predictor.

pred[, "id"] <- 0

pred["id", ] <- 0

\# Predictive mean matching for incomplete continuous outcomes

meth[followup_vars] <- "pmm"

\# Run multiple imputation.

\# Allocation, facility, sex, baseline values, and the other outcome

\# measurements remain available as predictors through pred.

imp <- mice(

dat,

m = M,

maxit = MAXIT,

method = meth,

predictorMatrix = pred,

seed = 500,

printFlag = TRUE

)

\# Save the complete MICE object so the analysis can be reproduced

saveRDS(imp, file = "primary_MI_m50.rds")

\# Check whether MICE logged any collinearity or other events

if (!is.null(imp$loggedEvents)) {

cat("\nMICE logged events:\n")

print(imp$loggedEvents)

} else {

cat("\nNo MICE logged events were recorded.\n")

}

\# ------------------------------------------------------------

\# 4. MICE diagnostics

\# ------------------------------------------------------------

\# Trace plots

png(

"MI_traceplots.png",

width = 1800, height = 1400, res = 150

)

plot(imp)

dev.off()

\# Density plots for each outcome family

outcome_names <- c("GARS", "LSAS", "SPIN", "PHQ", "GAD")

for (outcome in outcome_names) {

vars <- c(

paste0("post", outcome),

paste0("three", outcome),

paste0("six", outcome)

)

png(

paste0("MI_density_", outcome, ".png"),

width = 1800, height = 1100, res = 150

)

print(

densityplot(

imp,

as.formula(

paste("~", paste(vars, collapse = " + "))

)

)

)

dev.off()

}

\# ------------------------------------------------------------

\# 5. Create completed datasets

\# ------------------------------------------------------------

completed_datasets <- lapply(

seq_len(imp$m),

function(i) complete(imp, action = i)

)

\# ------------------------------------------------------------

\# 6. Functions for the LMM

\# ------------------------------------------------------------

make_long_data <- function(d, outcome) {

pre_var	<- paste0("pre", outcome)

post_var <- paste0("post", outcome)

three_var <- paste0("three", outcome)

six_var	<- paste0("six", outcome)

d %>%

transmute(

id,

allocation,

facility,

sex,

baseline = .data[[pre_var]],

Post = .data[[post_var]] - .data[[pre_var]],

`3M` = .data[[three_var]] - .data[[pre_var]],

`6M` = .data[[six_var]] - .data[[pre_var]]

) %>%

pivot_longer(

cols = c(Post, `3M`, `6M`),

names_to = "time",

values_to = "change"

) %>%

mutate(

time = factor(time, levels = c("Post", "3M", "6M")),

allocation = factor(

allocation,

levels = c("Control", "BookCBT", "WebCBT")

),

id = factor(id),

facility = factor(facility),

sex = factor(sex)

)

}

fit_lmm <- function(d, outcome) {

d_long <- make_long_data(d, outcome)

lmer(

change ~ allocation * time + baseline + sex + facility +

(1 | id),

data = d_long,

REML = TRUE,

control = lmerControl(

optimizer = "bobyqa",

optCtrl = list(maxfun = 100000)

)

)

}

\# ------------------------------------------------------------

\# 7. Fit the same LMM in every imputed dataset

\# ------------------------------------------------------------

fit_lists <- list()

mira_models <- list()

for (outcome in outcome_names) {

cat("\n====================================================\n")

cat("Fitting outcome:", outcome, "\n")

cat("====================================================\n")

fits <- lapply(

completed_datasets,

fit_lmm,

outcome = outcome

)

fit_lists[[outcome]] <- fits

mira_models[[outcome]] <- mice::as.mira(fits)

}

\# ------------------------------------------------------------

\# 8. Model convergence / singular-fit diagnostics

\# ------------------------------------------------------------

model_diagnostics <- bind_rows(

lapply(outcome_names, function(outcome) {

fits <- fit_lists[[outcome]]

bind_rows(

lapply(seq_along(fits), function(i) {

fit <- fits[[i]]

conv_msg <- fit@optinfo$conv$lme4$messages

if (is.null(conv_msg)) {

conv_msg <- NA_character_

} else {

conv_msg <- paste(conv_msg, collapse = " | ")

}

data.frame(

outcome = outcome,

imputation = i,

singular = isSingular(fit, tol = 1e-4),

convergence_message = conv_msg

)

})

)

})

)

cat("\nModel diagnostic summary:\n")

print(

model_diagnostics %>%

group_by(outcome) %>%

summarise(

n_singular = sum(singular),

n_convergence_messages = sum(!is.na(convergence_message)),

.groups = "drop"

)

)

\# ------------------------------------------------------------

\# 9. Residual diagnostics

\#	Use the first imputed dataset as a representative model.

\# ------------------------------------------------------------

for (outcome in outcome_names) {

fit <- fit_lists[[outcome]][[1]]

png(

paste0("LMM_diagnostics_", outcome, ".png"),

width = 1600, height = 750, res = 150

)

par(mfrow = c(1, 2))

plot(

fitted(fit),

resid(fit),

xlab = "Fitted values",

ylab = "Conditional residuals",

main = paste0(outcome, ": residuals vs fitted")

)

abline(h = 0, lty = 2)

qqnorm(

resid(fit),

main = paste0(outcome, ": normal Q-Q plot")

)

qqline(resid(fit))

par(mfrow = c(1, 1))

dev.off()

}

\# ------------------------------------------------------------

\# 10. Rubin-pooled estimated marginal means and Dunnett contrasts

\# ------------------------------------------------------------

\# Current emmeans supports mice::mira objects and combines model

\# estimates/covariances using multiple-imputation pooling rules.

all_contrasts <- list()

all_joint_tests <- list()

all_fixed_effects <- list()

for (outcome in outcome_names) {

fit_mira <- mira_models[[outcome]]

\# Optional: pooled fixed-effect coefficients

pooled_fixed <- mice::pool(fit_mira)

fixed_df <- summary(

pooled_fixed,

conf.int = TRUE,

conf.level = 0.95

)

fixed_df$outcome <- outcome

all_fixed_effects[[outcome]] <- fixed_df

\# Type-III-like joint tests based on pooled EMMs.

\# IMPORTANT:

\# Under the present change-score model, allocation:time has 4 numerator df

\# (2 treatment contrasts x 2 time contrasts), not 6.

joint_df <- as.data.frame(

emmeans::joint_tests(fit_mira)

)

joint_df$outcome <- outcome

all_joint_tests[[outcome]] <- joint_df

\# Adjusted marginal means by treatment at each follow-up time

emm <- emmeans::emmeans(

fit_mira,

~ allocation | time

)

\# Each active intervention vs Control.

\# "dunnett" is the treatment-vs-control contrast family in emmeans.

\# Its default multiplicity adjustment is Dunnett-type.

dun <- emmeans::contrast(

emm,

method = "dunnett",

ref = 1,

by = "time"

)

dun_df <- as.data.frame(

summary(

dun,

infer = c(TRUE, TRUE),

level = 0.95

)

)

dun_df$outcome <- outcome

all_contrasts[[outcome]] <- dun_df

}

contrast_results <- bind_rows(all_contrasts)

joint_tests_results <- bind_rows(all_joint_tests)

pooled_fixed_effects <- bind_rows(all_fixed_effects)

\# ------------------------------------------------------------

\# 11. Calculate Hedges' g from the pooled adjusted MD

\# ------------------------------------------------------------

\# Baseline scores are complete, so the pooled baseline SD is calculated

\# directly from the original master dataset.

\#

\# Hedges' g =

\#	J * (pooled adjusted mean difference / pooled baseline SD)

\#

\# The CI is obtained by applying the same positive scaling constant

\# to the confidence limits of the pooled adjusted mean difference.

group_n <- table(dat$allocation)

baseline_sd_table <- bind_rows(

lapply(outcome_names, function(outcome) {

pre_var <- paste0("pre", outcome)

dat %>%

group_by(allocation) %>%

summarise(

outcome = outcome,

n = n(),

baseline_mean = mean(.data[[pre_var]], na.rm = TRUE),

baseline_sd = sd(.data[[pre_var]], na.rm = TRUE),

.groups = "drop"

)

})

)

get_baseline_sd <- function(outcome, group_name) {

baseline_sd_table %>%

filter(

outcome == !!outcome,

allocation == !!group_name

) %>%

pull(baseline_sd)

}

get_group_n <- function(group_name) {

as.numeric(group_n[group_name])

}

calculate_g_row <- function(outcome, comparison, estimate, lower, upper) {

if (grepl("^BookCBT", comparison)) {

treatment <- "BookCBT"

comparison_label <- "Bib vs WLC"

} else if (grepl("^WebCBT", comparison)) {

treatment <- "WebCBT"

comparison_label <- "DSI vs WLC"

} else {

stop("Unexpected contrast label: ", comparison)

}

n1 <- get_group_n(treatment)

n0 <- get_group_n("Control")

sd1 <- get_baseline_sd(outcome, treatment)

sd0 <- get_baseline_sd(outcome, "Control")

pooled_baseline_sd <- sqrt(

((n1 - 1) * sd1^2 + (n0 - 1) * sd0^2) /

(n1 + n0 - 2)

)

\# Small-sample bias correction

J <- 1 - 3 / (4 * (n1 + n0) - 9)

data.frame(

comparison_label = comparison_label,

pooled_baseline_sd = pooled_baseline_sd,

J = J,

hedges_g = J * estimate / pooled_baseline_sd,

g_lower = J * lower / pooled_baseline_sd,

g_upper = J * upper / pooled_baseline_sd

)

}

table2_long <- bind_rows(

lapply(seq_len(nrow(contrast_results)), function(i) {

row <- contrast_results[i, ]

g_info <- calculate_g_row(

outcome = row$outcome,

comparison = as.character(row$contrast),

estimate = row$estimate,

lower = row$lower.CL,

upper = row$upper.CL

)

data.frame(

outcome = row$outcome,

time = as.character(row$time),

comparison = g_info$comparison_label,

adjusted_MD = row$estimate,

MD_lower = row$lower.CL,

MD_upper = row$upper.CL,

SE = row$SE,

df = row$df,

t_ratio = row$t.ratio,

p_value_Dunnett = row$p.value,

pooled_baseline_SD = g_info$pooled_baseline_sd,

Hedges_g = g_info$hedges_g,

g_lower = g_info$g_lower,

g_upper = g_info$g_upper

)

})

)

\# Order rows as in the manuscript

table2_long <- table2_long %>%

mutate(

outcome = factor(

outcome,

levels = c("GARS", "LSAS", "SPIN", "PHQ", "GAD")

),

time = factor(

time,

levels = c("Post", "3M", "6M")

),

comparison = factor(

comparison,

levels = c("Bib vs WLC", "DSI vs WLC")

)

) %>%

arrange(outcome, time, comparison)

\# Formatted strings for direct manuscript use

table2_formatted_long <- table2_long %>%

mutate(

`MD (95% CI)` = sprintf(

"%.2f (%.2f, %.2f)",

adjusted_MD, MD_lower, MD_upper

),

`Hedges' g (95% CI)` = sprintf(

"%.2f (%.2f, %.2f)",

Hedges_g, g_lower, g_upper

),

`Dunnett-adjusted P` = ifelse(

p_value_Dunnett < 0.001,

"<0.001",

sprintf("%.3f", p_value_Dunnett)

)

) %>%

select(

outcome, time, comparison,

`MD (95% CI)`,

`Hedges' g (95% CI)`,

`Dunnett-adjusted P`

)

\# Wide version resembling Table 2

table2_wide <- table2_formatted_long %>%

pivot_wider(

names_from = comparison,

values_from = c(

`MD (95% CI)`,

`Hedges' g (95% CI)`,

`Dunnett-adjusted P`

)

) %>%

arrange(outcome, time)

\# ------------------------------------------------------------

\# 12. Extract group x time pooled joint tests

\# ------------------------------------------------------------

interaction_tests <- joint_tests_results %>%

filter(`model term` == "allocation:time")

cat("\nPooled allocation x time tests:\n")

print(interaction_tests)

cat("\nFinal Table 2 results (long format):\n")

print(table2_formatted_long)

View(table2_formatted_long)

print(

dplyr::as_tibble(table2_formatted_long),

n = Inf,

width = Inf

)

\# ------------------------------------------------------------

\# 13. Export all results

\# ------------------------------------------------------------

output_list <- list(

Table2_long_numeric = as.data.frame(table2_long),

Table2_formatted_long = as.data.frame(table2_formatted_long),

Table2_wide = as.data.frame(table2_wide),

Interaction_tests = as.data.frame(interaction_tests),

All_joint_tests = as.data.frame(joint_tests_results),

Pooled_fixed_effects = as.data.frame(pooled_fixed_effects),

Baseline_SD = as.data.frame(baseline_sd_table),

Missingness = as.data.frame(missingness_summary),

Model_diagnostics = as.data.frame(model_diagnostics)

)

write_xlsx(

output_list,

path = "Primary_MI_LMM_results.xlsx"

)

\# Save the fitted MI model objects for later checking

saveRDS(

mira_models,

file = "Primary_MI_LMM_mira_models.rds"

)

\# Save session information for reproducibility

capture.output(

sessionInfo(),

file = "R_sessionInfo.txt"

)

cat("\n====================================================\n")

cat("Analysis completed.\n")

cat("Main output: Primary_MI_LMM_results.xlsx\n")

cat("MICE object: primary_MI_m50.rds\n")

cat("Pooled model objects: Primary_MI_LMM_mira_models.rds\n")

cat("Diagnostics: MI_traceplots.png, MI_density_*.png, LMM_diagnostics_*.png\n")

cat("====================================================\n")

\# ============================================================

\# Export diagnostic plots for the final GARS-J mixed model

\# ============================================================

\# Final model:

\#	change ~ allocation * time + baseline + sex + facility + (1 | id)

\#

\# This script uses the FIRST of 50 imputed datasets only for the

\# representative Appendix 4 plots. It also creates a PDF containing

\# diagnostics for all imputations if desired.

\#

\# Required packages:

\#	mice, lme4, ggplot2, dplyr, tidyr

\# ============================================================

library(mice)

library(lme4)

library(ggplot2)

library(dplyr)

library(tidyr)

\# ------------------------------------------------------------

\# 1. Obtain the mids object

\# ------------------------------------------------------------

\# If your imputed object is already in the R session as `imp`,

\# this section uses it.

\# Otherwise, save your final mids object as "primary_MI_m50.rds"

\# in the working directory, and the script will read it.

if (!exists("imp")) {

if (file.exists("primary_MI_m50.rds")) {

imp <- readRDS("primary_MI_m50.rds")

} else {

stop(

"No object named `imp` was found, and primary_MI_m50.rds ",

"was not found in the working directory.\n",

"Please load your final mice::mids object and name it `imp`, ",

"or save it as primary_MI_m50.rds."

)

}

}

if (!inherits(imp, "mids")) {

stop("`imp` must be a mice::mids object.")

}

\# ------------------------------------------------------------

\# 2. Variable names

\# ------------------------------------------------------------

\# Edit ONLY this block if your column names differ.

id_var	<- "id"

group_var	<- "allocation"

sex_var	<- "sex"

facility_var <- "facility"

pre_var	<- "preGARS"

post_var	<- "postGARS"

m3_var	<- "threeGARS"

m6_var	<- "sixGARS"

required_vars <- c(

id_var, group_var, sex_var, facility_var,

pre_var, post_var, m3_var, m6_var

)

missing_vars <- setdiff(required_vars, names(complete(imp, 1)))

if (length(missing_vars) > 0) {

stop(

"These variables were not found:\n ",

paste(missing_vars, collapse = ", "),

"\n\nPlease edit the variable-name block near the top of the script."

)

}

\# ------------------------------------------------------------

\# 3. Function to reshape one completed dataset and fit final model

\# ------------------------------------------------------------

fit_gars_model <- function(dat) {

long <- dat %>%

transmute(

id	= .data[[id_var]],

allocation = factor(.data[[group_var]]),

sex	= factor(.data[[sex_var]]),

facility	= factor(.data[[facility_var]]),

baseline	= .data[[pre_var]],

Post	= .data[[post_var]],

`3M`	= .data[[m3_var]],

`6M`	= .data[[m6_var]]

) %>%

pivot_longer(

cols = c(Post, `3M`, `6M`),

names_to = "time",

values_to = "score"

) %>%

mutate(

time = factor(time, levels = c("Post", "3M", "6M")),

change = score - baseline

)

fit <- lmer(

change ~ allocation * time + baseline + sex + facility + (1 | id),

data = long,

REML = TRUE

)

list(data = long, fit = fit)

}

\# ------------------------------------------------------------

\# 4. Representative diagnostics: first imputed dataset

\# ------------------------------------------------------------

d1 <- complete(imp, 1)

obj1 <- fit_gars_model(d1)

fit1 <- obj1$fit

diag_df <- data.frame(

fitted = fitted(fit1),

std_resid = resid(fit1) / sigma(fit1)

)

\# Residuals-versus-fitted plot

p_resid <- ggplot(diag_df, aes(x = fitted, y = std_resid)) +

geom_point(alpha = 0.55, size = 1.5) +

geom_hline(yintercept = 0, linetype = 2, linewidth = 0.5) +

geom_smooth(method = "loess", se = FALSE, linewidth = 0.7) +

labs(

x = "Fitted values",

y = "Standardized conditional residuals"

) +

theme_classic(base_size = 12)

ggsave(

"Appendix4_GARS_residuals_vs_fitted.png",

p_resid, width = 6.5, height = 5.0, dpi = 600

)

ggsave(

"Appendix4_GARS_residuals_vs_fitted.pdf",

p_resid, width = 6.5, height = 5.0

)

ggsave(

"Appendix4_GARS_residuals_vs_fitted.tiff",

p_resid, width = 6.5, height = 5.0, dpi = 600,

compression = "lzw"

)

\# Q-Q plot

p_qq <- ggplot(diag_df, aes(sample = std_resid)) +

stat_qq(size = 1.5, alpha = 0.65) +

stat_qq_line(linewidth = 0.7) +

labs(

x = "Theoretical quantiles",

y = "Standardized conditional residuals"

) +

theme_classic(base_size = 12)

ggsave(

"Appendix4_GARS_QQ_plot.png",

p_qq, width = 6.5, height = 5.0, dpi = 600

)

ggsave(

"Appendix4_GARS_QQ_plot.pdf",

p_qq, width = 6.5, height = 5.0

)

ggsave(

"Appendix4_GARS_QQ_plot.tiff",

p_qq, width = 6.5, height = 5.0, dpi = 600,

compression = "lzw"

)

\# ------------------------------------------------------------

\# 5. QC across all imputations

\# ------------------------------------------------------------

qc <- data.frame(

imputation = seq_len(imp$m),

singular = NA,

convergence_message = NA_character_

)

pdf(

"QC_GARS_diagnostics_all_imputations.pdf",

width = 10, height = 5.5

)

for (i in seq_len(imp$m)) {

di <- complete(imp, i)

oi <- fit_gars_model(di)

fi <- oi$fit

qc$singular[i] <- isSingular(fi, tol = 1e-4)

conv_msg <- fi@optinfo$conv$lme4$messages

qc$convergence_message[i] <- if (is.null(conv_msg)) {

""

} else {

paste(conv_msg, collapse = " | ")

}

dd <- data.frame(

fitted = fitted(fi),

std_resid = resid(fi) / sigma(fi)

)

par(mfrow = c(1, 2))

plot(

dd$fitted, dd$std_resid,

pch = 16, cex = 0.55,

xlab = "Fitted values",

ylab = "Standardized conditional residuals",

main = paste0("Residuals vs fitted: imputation ", i)

)

abline(h = 0, lty = 2)

qqnorm(

dd$std_resid,

pch = 16, cex = 0.55,

main = paste0("Q-Q plot: imputation ", i)

)

qqline(dd$std_resid, lty = 2)

}

dev.off()

write.csv(

qc,

"QC_GARS_model_diagnostics.csv",

row.names = FALSE

)

\# ------------------------------------------------------------

\# 6. Console summary

\# ------------------------------------------------------------

cat("\nCompleted.\n")

cat("Representative Appendix 4 figures:\n")

cat(" Appendix4_GARS_residuals_vs_fitted.png\n")

cat(" Appendix4_GARS_QQ_plot.png\n\n")

cat("QC files:\n")

cat(" QC_GARS_diagnostics_all_imputations.pdf\n")

cat(" QC_GARS_model_diagnostics.csv\n\n")

cat(

"Singular fits:",

sum(qc$singular, na.rm = TRUE),

"of",

nrow(qc),

"\n"

)

cat(

"Fits with convergence messages:",

sum(nzchar(qc$convergence_message)),

"of",

nrow(qc),

"\n"

)

\# ============================================================

\# CLINICALLY SIGNIFICANT OUTCOMES AFTER MULTIPLE IMPUTATION

\#

\# Uses the SAME MICE object as the continuous primary analysis.

\#

\# Workflow:

\#	1. Load the 50-imputation mids object

\#	2. Complete each imputed dataset

\#	3. Derive Response / Remission / Recovery / Deterioration

\#	deterministically within EACH imputed dataset

\#	4. Pool:

\#	- group proportions

\#	- risk ratios (modified Poisson + robust SE)

\#	- odds ratios (logistic regression)

\#	- absolute risk reduction for deterioration

\#	5. Calculate NNTB/NNTH from the pooled ARR point estimate

\#	6. Export manuscript/supplement-ready tables and Figure 2 data

\#

\# Definitions:

\#	Response:

\#	>=28% decrease from baseline in LSAS total score

\#	Remission:

\#	LSAS total score <35

\#	Recovery:

\#	SPIN total score <19 AND PHQ-9 total score <10

\#	Deterioration:

\#	>=28% increase from baseline in LSAS total score

\#

\# IMPORTANT:

\# - Do NOT impute the binary outcomes themselves.

\# - They are derived after imputation from the imputed continuous scales.

\# - There is no single integer event count after multiple imputation.

\#	Therefore manuscript tables should preferentially report pooled

\#	percentages rather than a single n (%).

\# ============================================================

rm(list = ls())

gc()

set.seed(500)

\# ------------------------------------------------------------

\# 0. User settings

\# ------------------------------------------------------------

\# Use the MICE object saved from the FINAL primary continuous analysis.

IMP_FILE <- "primary_MI_m50.rds"

\# Change this only if you intentionally run the same pipeline on another

\# imputation object (e.g., a sensitivity-analysis mids object).

ANALYSIS_LABEL <- "Primary"

OUTPUT_PREFIX <- paste0(ANALYSIS_LABEL, "_ClinicallySignificant_MI")

CONF_LEVEL <- 0.95

\# ------------------------------------------------------------

\# 1. Packages

\# ------------------------------------------------------------

packages <- c(

"mice",

"dplyr",

"tidyr",

"purrr",

"sandwich",

"ggplot2",

"writexl"

)

not_installed <- packages[

!vapply(packages, requireNamespace, logical(1), quietly = TRUE)

]

if (length(not_installed) > 0) {

install.packages(not_installed)

}

library(mice)

library(dplyr)

library(tidyr)

library(purrr)

library(sandwich)

library(ggplot2)

library(writexl)

\# ------------------------------------------------------------

\# 2. Load the SAME MICE object used for the main analysis

\# ------------------------------------------------------------

if (!file.exists(IMP_FILE)) {

message(

IMP_FILE,

" was not found in the working directory.\n",

"Please choose the correct .rds MICE object manually."

)

IMP_FILE <- file.choose()

}

imp <- readRDS(IMP_FILE)

if (!inherits(imp, "mids")) {

stop("The selected object is not a mice 'mids' object.")

}

cat("\n============================================\n")

cat("Loaded MICE object:", IMP_FILE, "\n")

cat("Number of imputations:", imp$m, "\n")

cat("============================================\n")

if (imp$m != 50) {

warning(

"This MICE object contains ", imp$m,

" imputations rather than 50. ",

"Confirm that this is the intended FINAL imputation object."

)

}

\# ------------------------------------------------------------

\# 3. Create all completed datasets

\# ------------------------------------------------------------

completed_datasets <- lapply(

seq_len(imp$m),

function(i) complete(imp, action = i)

)

\# ------------------------------------------------------------

\# 4. Standardise allocation coding

\# ------------------------------------------------------------

normalise_allocation <- function(x) {

if (is.factor(x) &&

all(c("Control", "BookCBT", "WebCBT") %in% levels(x))) {

return(

factor(

x,

levels = c("Control", "BookCBT", "WebCBT")

)

)

}

x_chr <- as.character(x)

if (all(na.omit(unique(x_chr)) %in% c("1", "2", "3"))) {

return(

factor(

x_chr,

levels = c("1", "2", "3"),

labels = c("Control", "BookCBT", "WebCBT")

)

)

}

if (all(

na.omit(unique(x_chr)) %in%

c("Control", "BookCBT", "WebCBT")

)) {

return(

factor(

x_chr,

levels = c("Control", "BookCBT", "WebCBT")

)

)

}

stop(

"allocation coding was not recognised. ",

"Expected 1/2/3 or Control/BookCBT/WebCBT."

)

}

\# ------------------------------------------------------------

\# 5. Derive clinically significant binary outcomes

\# ------------------------------------------------------------

make_binary_long <- function(d, imputation_number) {

required <- c(

"id", "allocation",

"preLSAS",

"postLSAS", "threeLSAS", "sixLSAS",

"postSPIN", "threeSPIN", "sixSPIN",

"postPHQ", "threePHQ", "sixPHQ"

)

missing_vars <- setdiff(required, names(d))

if (length(missing_vars) > 0) {

stop(

"Required variables are missing: ",

paste(missing_vars, collapse = ", ")

)

}

d <- d %>%

mutate(

allocation = normalise_allocation(allocation)

)

if (any(d$preLSAS <= 0, na.rm = TRUE)) {

stop(

"At least one participant has preLSAS <= 0. ",

"Response/deterioration based on percentage change is undefined."

)

}

post <- d %>%

transmute(

id,

allocation,

imputation = imputation_number,

time = "Post",

Response =

as.integer(

(preLSAS - postLSAS) / preLSAS >= 0.28

),

Remission =

as.integer(

postLSAS < 35

),

Recovery =

as.integer(

postSPIN < 19 &

postPHQ < 10

),

Deterioration =

as.integer(

(postLSAS - preLSAS) / preLSAS >= 0.28

)

)

three <- d %>%

transmute(

id,

allocation,

imputation = imputation_number,

time = "3M",

Response =

as.integer(

(preLSAS - threeLSAS) / preLSAS >= 0.28

),

Remission =

as.integer(

threeLSAS < 35

),

Recovery =

as.integer(

threeSPIN < 19 &

threePHQ < 10

),

Deterioration =

as.integer(

(threeLSAS - preLSAS) / preLSAS >= 0.28

)

)

six <- d %>%

transmute(

id,

allocation,

imputation = imputation_number,

time = "6M",

Response =

as.integer(

(preLSAS - sixLSAS) / preLSAS >= 0.28

),

Remission =

as.integer(

sixLSAS < 35

),

Recovery =

as.integer(

sixSPIN < 19 &

sixPHQ < 10

),

Deterioration =

as.integer(

(sixLSAS - preLSAS) / preLSAS >= 0.28

)

)

bind_rows(post, three, six) %>%

pivot_longer(

cols = c(

Response,

Remission,

Recovery,

Deterioration

),

names_to = "endpoint",

values_to = "event"

) %>%

mutate(

endpoint = factor(

endpoint,

levels = c(

"Response",

"Remission",

"Recovery",

"Deterioration"

)

),

time = factor(

time,

levels = c(

"Post",

"3M",

"6M"

)

),

allocation = factor(

allocation,

levels = c(

"Control",

"BookCBT",

"WebCBT"

)

)

)

}

binary_long_list <- Map(

make_binary_long,

completed_datasets,

seq_along(completed_datasets)

)

binary_all <- bind_rows(binary_long_list)

if (anyNA(binary_all$event)) {

stop(

"NA values remain in derived binary outcomes. ",

"Inspect the imputation object and definitions."

)

}

\# ------------------------------------------------------------

\# 6. Helper: Rubin pooling for one scalar estimate

\# ------------------------------------------------------------

pool_scalar_result <- function(

Q,

U,

n,

k,

conf.level = 0.95) {

pooled <- mice::pool.scalar(

Q = Q,

U = U,

n = n,

k = k,

rule = "rubin1987"

)

estimate <- pooled$qbar

se <- sqrt(pooled$t)

df <- pooled$df

alpha <- 1 - conf.level

crit <- qt(1 - alpha / 2, df = df)

lower <- estimate - crit * se

upper <- estimate + crit * se

p_value <- 2 * pt(

abs(estimate / se),

df = df,

lower.tail = FALSE

)

data.frame(

estimate = estimate,

SE = se,

df = df,

lower = lower,

upper = upper,

p_value = p_value,

FMI = pooled$fmi

)

}

\# ------------------------------------------------------------

\# 7. Pooled group proportions

\# ------------------------------------------------------------

prop_by_imp <- binary_all %>%

group_by(

imputation,

endpoint,

time,

allocation

) %>%

summarise(

n = n(),

events = sum(event),

proportion = mean(event),

within_var =

proportion * (1 - proportion) / n,

.groups = "drop"

)

pooled_proportions <- prop_by_imp %>%

group_by(

endpoint,

time,

allocation

) %>%

group_modify(function(.x, .y) {

n_group <- unique(.x$n)

if (length(n_group) != 1) {

stop("Group denominator differs across imputations.")

}

pooled <- pool_scalar_result(

Q = .x$proportion,

U = .x$within_var,

n = n_group,

k = 1,

conf.level = CONF_LEVEL

)

data.frame(

denominator = n_group,

mean_imputed_events = mean(.x$events),

pooled_proportion = pooled$estimate,

lower = max(0, pooled$lower),

upper = min(1, pooled$upper),

SE = pooled$SE,

df = pooled$df,

FMI = pooled$FMI

)

}) %>%

ungroup() %>%

mutate(

pooled_percent = 100 * pooled_proportion,

lower_percent = 100 * lower,

upper_percent = 100 * upper

)

\# ------------------------------------------------------------

\# 8. Risk ratios:

\#	modified Poisson regression + robust sandwich SE

\# ------------------------------------------------------------

fit_rr_one <- function(d_sub) {

d_sub <- d_sub %>%

mutate(

allocation = factor(

allocation,

levels = c(

"Control",

"BookCBT",

"WebCBT"

)

)

)

fit <- glm(

event ~ allocation,

family = poisson(link = "log"),

data = d_sub

)

V <- sandwich::vcovHC(

fit,

type = "HC0"

)

b <- coef(fit)

terms <- c(

"allocationBookCBT",

"allocationWebCBT"

)

data.frame(

comparison = c(

"Bib vs WLC",

"DSI vs WLC"

),

estimate_log = b[terms],

variance_log = diag(V)[terms],

n_total = nrow(d_sub)

)

}

rr_by_imp <- bind_rows(

lapply(

seq_along(binary_long_list),

function(i) {

d_i <- binary_long_list[[i]]

bind_rows(

lapply(

levels(d_i$endpoint),

function(endpoint_i) {

bind_rows(

lapply(

levels(d_i$time),

function(time_i) {

d_sub <- d_i %>%

filter(

endpoint == endpoint_i,

time == time_i

)

out <- fit_rr_one(d_sub)

out %>%

mutate(

imputation = i,

endpoint = endpoint_i,

time = time_i

)

}

)

)

}

)

)

}

)

)

pooled_RR <- rr_by_imp %>%

group_by(

endpoint,

time,

comparison

) %>%

group_modify(function(.x, .y) {

n_total <- unique(.x$n_total)

pooled <- pool_scalar_result(

Q = .x$estimate_log,

U = .x$variance_log,

n = n_total,

k = 3,

conf.level = CONF_LEVEL

)

data.frame(

logRR = pooled$estimate,

SE_logRR = pooled$SE,

df = pooled$df,

RR = exp(pooled$estimate),

RR_lower = exp(pooled$lower),

RR_upper = exp(pooled$upper),

p_value = pooled$p_value,

FMI = pooled$FMI

)

}) %>%

ungroup()

\# ------------------------------------------------------------

\# 9. Odds ratios:

\#	logistic regression + Rubin pooling

\# ------------------------------------------------------------

fit_or_one <- function(d_sub) {

d_sub <- d_sub %>%

mutate(

allocation = factor(

allocation,

levels = c(

"Control",

"BookCBT",

"WebCBT"

)

)

)

fit <- glm(

event ~ allocation,

family = binomial(link = "logit"),

data = d_sub

)

b <- coef(fit)

V <- vcov(fit)

terms <- c(

"allocationBookCBT",

"allocationWebCBT"

)

if (any(!is.finite(b[terms])) ||

any(!is.finite(diag(V)[terms]))) {

stop(

"Non-finite logistic coefficient/variance encountered. ",

"Check for complete or quasi-complete separation."

)

}

data.frame(

comparison = c(

"Bib vs WLC",

"DSI vs WLC"

),

estimate_log = b[terms],

variance_log = diag(V)[terms],

n_total = nrow(d_sub)

)

}

or_by_imp <- bind_rows(

lapply(

seq_along(binary_long_list),

function(i) {

d_i <- binary_long_list[[i]]

bind_rows(

lapply(

levels(d_i$endpoint),

function(endpoint_i) {

bind_rows(

lapply(

levels(d_i$time),

function(time_i) {

d_sub <- d_i %>%

filter(

endpoint == endpoint_i,

time == time_i

)

out <- fit_or_one(d_sub)

out %>%

mutate(

imputation = i,

endpoint = endpoint_i,

time = time_i

)

}

)

)

}

)

)

}

)

)

pooled_OR <- or_by_imp %>%

group_by(

endpoint,

time,

comparison

) %>%

group_modify(function(.x, .y) {

n_total <- unique(.x$n_total)

pooled <- pool_scalar_result(

Q = .x$estimate_log,

U = .x$variance_log,

n = n_total,

k = 3,

conf.level = CONF_LEVEL

)

data.frame(

logOR = pooled$estimate,

SE_logOR = pooled$SE,

df = pooled$df,

OR = exp(pooled$estimate),

OR_lower = exp(pooled$lower),

OR_upper = exp(pooled$upper),

p_value = pooled$p_value,

FMI = pooled$FMI

)

}) %>%

ungroup()

\# ------------------------------------------------------------

\# 10. Absolute risk reduction for DETERIORATION + NNT

\# ------------------------------------------------------------

fit_arr_one <- function(d_sub) {

d_sub <- d_sub %>%

mutate(

allocation = factor(

allocation,

levels = c(

"Control",

"BookCBT",

"WebCBT"

)

)

)

fit <- lm(

event ~ allocation,

data = d_sub

)

V <- sandwich::vcovHC(

fit,

type = "HC0"

)

b <- coef(fit)

terms <- c(

"allocationBookCBT",

"allocationWebCBT"

)

data.frame(

comparison = c(

"Bib vs WLC",

"DSI vs WLC"

),

ARR = -b[terms],

ARR_variance = diag(V)[terms],

n_total = nrow(d_sub)

)

}

arr_by_imp <- bind_rows(

lapply(

seq_along(binary_long_list),

function(i) {

d_i <- binary_long_list[[i]] %>%

filter(endpoint == "Deterioration")

bind_rows(

lapply(

levels(d_i$time),

function(time_i) {

d_sub <- d_i %>%

filter(time == time_i)

out <- fit_arr_one(d_sub)

out %>%

mutate(

imputation = i,

time = time_i

)

}

)

)

}

)

)

pooled_ARR <- arr_by_imp %>%

group_by(

time,

comparison

) %>%

group_modify(function(.x, .y) {

n_total <- unique(.x$n_total)

pooled <- pool_scalar_result(

Q = .x$ARR,

U = .x$ARR_variance,

n = n_total,

k = 3,

conf.level = CONF_LEVEL

)

arr <- pooled$estimate

if (arr > 0) {

nnt_type <- "NNTB"

nnt <- 1 / arr

} else if (arr < 0) {

nnt_type <- "NNTH"

nnt <- 1 / abs(arr)

} else {

nnt_type <- "No effect"

nnt <- Inf

}

data.frame(

ARR = arr,

ARR_lower = pooled$lower,

ARR_upper = pooled$upper,

ARR_percent = 100 * arr,

ARR_lower_percent = 100 * pooled$lower,

ARR_upper_percent = 100 * pooled$upper,

SE = pooled$SE,

df = pooled$df,

p_value = pooled$p_value,

FMI = pooled$FMI,

NNT_type = nnt_type,

NNT = nnt

)

}) %>%

ungroup()

\# ------------------------------------------------------------

\# 11. Add deterioration pooled risks and RRR

\# ------------------------------------------------------------

deterioration_props <- pooled_proportions %>%

filter(endpoint == "Deterioration") %>%

select(

time,

allocation,

pooled_proportion,

pooled_percent

)

deterioration_wide <- deterioration_props %>%

select(

time,

allocation,

pooled_percent

) %>%

pivot_wider(

names_from = allocation,

values_from = pooled_percent

)

deterioration_rr <- pooled_RR %>%

filter(endpoint == "Deterioration") %>%

mutate(

RRR = 1 - RR,

RRR_percent = 100 * RRR

)

appendix11_numeric <- pooled_ARR %>%

left_join(

deterioration_rr %>%

select(

time,

comparison,

RR,

RR_lower,

RR_upper,

RRR,

RRR_percent

),

by = c("time", "comparison")

) %>%

left_join(

deterioration_wide,

by = "time"

) %>%

mutate(

intervention_percent = case_when(

comparison == "Bib vs WLC" ~ BookCBT,

comparison == "DSI vs WLC" ~ WebCBT,

TRUE ~ NA_real_

),

WLC_percent = Control

) %>%

select(

time,

comparison,

intervention_percent,

WLC_percent,

RR,

RR_lower,

RR_upper,

RRR,

RRR_percent,

ARR,

ARR_lower,

ARR_upper,

ARR_percent,

ARR_lower_percent,

ARR_upper_percent,

NNT_type,

NNT,

p_value,

FMI

)

\# ------------------------------------------------------------

\# 12. Display tables

\# ------------------------------------------------------------

group_display <- c(

"Control" = "WLC",

"BookCBT" = "Bib",

"WebCBT" = "DSI"

)

pooled_proportions_display <- pooled_proportions %>%

mutate(

Group = unname(

group_display[as.character(allocation)]

),

`Pooled % (95% CI)` = sprintf(

"%.1f (%.1f, %.1f)",

pooled_percent,

lower_percent,

upper_percent

)

) %>%

select(

endpoint,

time,

Group,

denominator,

`Pooled % (95% CI)`

)

rr_display <- pooled_RR %>%

mutate(

`RR (95% CI)` = sprintf(

"%.2f (%.2f, %.2f)",

RR,

RR_lower,

RR_upper

),

`P value` = ifelse(

p_value < 0.001,

"<0.001",

sprintf("%.3f", p_value)

)

) %>%

select(

endpoint,

time,

comparison,

`RR (95% CI)`,

`P value`

)

or_display <- pooled_OR %>%

mutate(

`OR (95% CI)` = sprintf(

"%.2f (%.2f, %.2f)",

OR,

OR_lower,

OR_upper

),

`P value` = ifelse(

p_value < 0.001,

"<0.001",

sprintf("%.3f", p_value)

)

) %>%

select(

endpoint,

time,

comparison,

`OR (95% CI)`,

`P value`

)

table_S3_RR <- pooled_proportions %>%

mutate(

Group = unname(

group_display[as.character(allocation)]

)

) %>%

select(

endpoint,

time,

Group,

pooled_percent

) %>%

left_join(

pooled_RR %>%

mutate(

Group = case_when(

comparison == "Bib vs WLC" ~ "Bib",

comparison == "DSI vs WLC" ~ "DSI"

)

) %>%

select(

endpoint,

time,

Group,

RR,

RR_lower,

RR_upper

),

by = c(

"endpoint",

"time",

"Group"

)

) %>%

mutate(

`Pooled %` = sprintf(

"%.1f",

pooled_percent

),

`RR (95% CI)` = ifelse(

Group == "WLC",

"Reference",

sprintf(

"%.2f (%.2f, %.2f)",

RR,

RR_lower,

RR_upper

)

)

) %>%

select(

endpoint,

time,

Group,

`Pooled %`,

`RR (95% CI)`

)

table_S4_OR <- pooled_proportions %>%

mutate(

Group = unname(

group_display[as.character(allocation)]

)

) %>%

select(

endpoint,

time,

Group,

pooled_percent

) %>%

left_join(

pooled_OR %>%

mutate(

Group = case_when(

comparison == "Bib vs WLC" ~ "Bib",

comparison == "DSI vs WLC" ~ "DSI"

)

) %>%

select(

endpoint,

time,

Group,

OR,

OR_lower,

OR_upper

),

by = c(

"endpoint",

"time",

"Group"

)

) %>%

mutate(

`Pooled %` = sprintf(

"%.1f",

pooled_percent

),

`OR (95% CI)` = ifelse(

Group == "WLC",

"Reference",

sprintf(

"%.2f (%.2f, %.2f)",

OR,

OR_lower,

OR_upper

)

)

) %>%

select(

endpoint,

time,

Group,

`Pooled %`,

`OR (95% CI)`

)

appendix11_display <- appendix11_numeric %>%

mutate(

`Deterioration, intervention (%)` =

sprintf("%.1f", intervention_percent),

`Deterioration, WLC (%)` =

sprintf("%.1f", WLC_percent),

`RR (95% CI)` =

sprintf(

"%.2f (%.2f, %.2f)",

RR,

RR_lower,

RR_upper

),

`ARR, % (95% CI)` =

sprintf(

"%.1f (%.1f, %.1f)",

ARR_percent,

ARR_lower_percent,

ARR_upper_percent

),

`NNT` =

ifelse(

is.finite(NNT),

paste0(

NNT_type,

" ",

sprintf("%.1f", NNT)

),

"∞"

)

) %>%

select(

time,

comparison,

`Deterioration, intervention (%)`,

`Deterioration, WLC (%)`,

`ARR, % (95% CI)`,

`NNT`,

`RR (95% CI)`

)

\# ------------------------------------------------------------

\# 13. Figure 2 data + preview

\# ------------------------------------------------------------

figure2_data <- pooled_proportions %>%

mutate(

Group = factor(

unname(

group_display[as.character(allocation)]

),

levels = c(

"WLC",

"Bib",

"DSI"

)

),

Outcome = factor(

as.character(endpoint),

levels = c(

"Response",

"Remission",

"Recovery",

"Deterioration"

)

),

Time = factor(

as.character(time),

levels = c(

"Post",

"3M",

"6M"

)

)

) %>%

transmute(

Outcome,

Time,

Group,

percentage = pooled_percent,

lower = lower_percent,

upper = upper_percent

)

fig2 <- ggplot(

figure2_data,

aes(

x = Time,

y = percentage,

fill = Group

)

) +

geom_col(

position = position_dodge(width = 0.8),

width = 0.72

) +

geom_errorbar(

aes(

ymin = lower,

ymax = upper

),

position = position_dodge(width = 0.8),

width = 0.18

) +

facet_wrap(

~ Outcome,

scales = "free_y"

) +

labs(

x = "Assessment time point",

y = "Pooled estimated proportion (%)",

fill = "Group"

) +

theme_classic(base_size = 12) +

theme(

legend.position = "bottom"

)

ggsave(

filename = paste0(

OUTPUT_PREFIX,

"_Figure2_preview.png"

),

plot = fig2,

width = 9,

height = 6.5,

dpi = 300

)

\# ------------------------------------------------------------

\# 14. Quality checks

\# ------------------------------------------------------------

denominator_check <- prop_by_imp %>%

group_by(

endpoint,

time,

allocation

) %>%

summarise(

min_n = min(n),

max_n = max(n),

.groups = "drop"

)

if (any(

denominator_check$min_n !=

denominator_check$max_n

)) {

warning(

"At least one group denominator differs across imputations."

)

}

effect_check <- data.frame(

RR_nonfinite =

sum(!is.finite(pooled_RR$RR)),

OR_nonfinite =

sum(!is.finite(pooled_OR$OR)),

ARR_nonfinite =

sum(!is.finite(pooled_ARR$ARR))

)

print(effect_check)

\# ------------------------------------------------------------

\# 15. Console output

\# ------------------------------------------------------------

cat("\n============================================\n")

cat("POOLED PROPORTIONS\n")

cat("============================================\n")

print(

as_tibble(pooled_proportions_display),

n = Inf,

width = Inf

)

cat("\n============================================\n")

cat("POOLED RISK RATIOS\n")

cat("============================================\n")

print(

as_tibble(rr_display),

n = Inf,

width = Inf

)

cat("\n============================================\n")

cat("POOLED ODDS RATIOS\n")

cat("============================================\n")

print(

as_tibble(or_display),

n = Inf,

width = Inf

)

cat("\n============================================\n")

cat("DETERIORATION ARR / NNT\n")

cat("============================================\n")

print(

as_tibble(appendix11_display),

n = Inf,

width = Inf

)

\# ------------------------------------------------------------

\# 16. Export to Excel

\# ------------------------------------------------------------

write_xlsx(

list(

Pooled_Proportions =

as.data.frame(pooled_proportions),

Pooled_Proportions_Display =

as.data.frame(pooled_proportions_display),

RR_Numeric =

as.data.frame(pooled_RR),

RR_Display =

as.data.frame(rr_display),

OR_Numeric =

as.data.frame(pooled_OR),

OR_Display =

as.data.frame(or_display),

Table_S3_RR =

as.data.frame(table_S3_RR),

Table_S4_OR =

as.data.frame(table_S4_OR),

Deterioration_ARR_NNT =

as.data.frame(appendix11_numeric),

Appendix11_Display =

as.data.frame(appendix11_display),

Figure2_Data =

as.data.frame(figure2_data),

Denominator_Check =

as.data.frame(denominator_check),

Effect_Check =

as.data.frame(effect_check)

),

path = paste0(

OUTPUT_PREFIX,

"_results.xlsx"

)

)

\# ------------------------------------------------------------

\# 17. Save pooled objects and session information

\# ------------------------------------------------------------

saveRDS(

list(

pooled_proportions = pooled_proportions,

pooled_RR = pooled_RR,

pooled_OR = pooled_OR,

pooled_ARR = pooled_ARR,

appendix11_numeric = appendix11_numeric,

figure2_data = figure2_data

),

file = paste0(

OUTPUT_PREFIX,

"_pooled_objects.rds"

)

)

capture.output(

sessionInfo(),

file = paste0(

OUTPUT_PREFIX,

"_sessionInfo.txt"

)

)

cat("\n============================================\n")

cat("ANALYSIS COMPLETE\n")

cat("============================================\n")

cat(

"\nMain Excel output:\n",

paste0(

OUTPUT_PREFIX,

"_results.xlsx"

),

"\n"

)

cat(

"\nFigure 2 preview:\n",

paste0(

OUTPUT_PREFIX,

"_Figure2_preview.png"

),

"\n"

)

cat("\nIMPORTANT:\n")

cat(

"- Use pooled percentages rather than a single integer n (%) in MI-based tables.\n"

)

cat(

"- RR estimates use modified Poisson regression with robust HC0 SEs.\n"

)

cat(

"- OR estimates use logistic regression.\n"

)

cat(

"- ARR is control risk minus intervention risk.\n"

)

cat(

"- NNT is a point estimate and should be interpreted together with the ARR 95% CI.\n"

)

\## Figure 2

library(ggplot2)

library(dplyr)

\# ------------------------------------------------------------

\# Figure 2

\# Clinically significant outcomes after multiple imputation

\# ------------------------------------------------------------

plot_data <- figure2_data %>%

mutate(

Outcome = factor(

Outcome,

levels = c(

"Response",

"Remission",

"Recovery",

"Deterioration"

)

),

Time = factor(

Time,

levels = c("Post", "3M", "6M")

),

Group = factor(

Group,

levels = c("Bib", "DSI", "WLC")

)

)

fig2 <- ggplot(

plot_data,

aes(

x = Time,

y = percentage,

group = Group,

shape = Group,

linetype = Group

)

) +

\# 95% CI

geom_errorbar(

aes(

ymin = lower,

ymax = upper

),

width = 0.12,

linewidth = 0.65

) +

\# Lines

geom_line(

linewidth = 0.8

) +

\# Points

geom_point(

size = 3.0,

stroke = 0.9

) +

\# Four panels

facet_wrap(

~ Outcome,

ncol = 2

) +

\# Monochrome shapes

scale_shape_manual(

values = c(

"Bib" = 17, # filled triangle

"DSI" = 15, # filled square

"WLC" = 1	# open circle

)

) +

\# Line types

scale_linetype_manual(

values = c(

"Bib" = "solid",

"DSI" = "dashed",

"WLC" = "dotted"

)

) +

\# Same y-axis across all four panels

scale_y_continuous(

breaks = seq(0, 50, by = 20),

expand = expansion(mult = c(0.02, 0.05))

) +

coord_cartesian(

ylim = c(0, 55)

) +

labs(

x = "Time",

y = "Proportion (%)",

shape = "Group",

linetype = "Group"

) +

theme_bw(

base_size = 12

) +

theme(

\# Facet headers

strip.background = element_rect(

fill = "grey85",

linewidth = 0.6

),

strip.text = element_text(

face = "bold",

size = 11

),

\# Panel

panel.border = element_rect(

linewidth = 0.6

),

panel.grid.minor = element_blank(),

panel.grid.major.x = element_line(

linewidth = 0.3

),

panel.grid.major.y = element_line(

linewidth = 0.3

),

\# Axes

axis.title = element_text(

size = 12

),

axis.text = element_text(

size = 10

),

\# Legend

legend.position = "bottom",

legend.direction = "horizontal",

legend.box = "horizontal",

legend.title = element_text(

size = 11

),

legend.text = element_text(

size = 10

),

\# Spacing

panel.spacing = unit(

0.15,

"cm"

),

plot.margin = margin(

8, 8, 8, 8

)

)

\# Display

print(fig2)

\# ------------------------------------------------------------

\# Save

\# ------------------------------------------------------------

\# High-resolution TIFF for journal submission

ggsave(

filename = "Figure2_clinically_significant_outcomes.tiff",

plot = fig2,

width = 170,

height = 175,

units = "mm",

dpi = 600,

compression = "lzw"

)

\# PDF for editing / manuscript preparation

ggsave(

filename = "Figure2_clinically_significant_outcomes.pdf",

plot = fig2,

width = 170,

height = 175,

units = "mm"

)

\## Q-Q plots

library(ggplot2)

library(dplyr)

d1 <- complete(imp, 1)

long1 <- d1 %>%

transmute(

id,

allocation,

sex,

facility,

baseline = preGARS,

Post = postGARS,

`3M` = threeGARS,

`6M` = sixGARS

) %>%

pivot_longer(

cols = c(Post, `3M`, `6M`),

names_to = "time",

values_to = "score"

) %>%

mutate(

time = factor(time, levels = c("Post", "3M", "6M")),

change = score - baseline

)

fit1 <- lmer(

change ~ allocation * time + baseline + sex + facility + (1 | id),

data = long1,

REML = TRUE

)

diag_df <- data.frame(

fitted = fitted(fit1),

std_resid = resid(fit1) / sigma(fit1)

)

p_resid <- ggplot(diag_df, aes(x = fitted, y = std_resid)) +

geom_point(alpha = 0.55, size = 1.5) +

geom_hline(yintercept = 0, linetype = 2, linewidth = 0.5) +

geom_smooth(method = "loess", se = FALSE, linewidth = 0.7) +

labs(

x = "Fitted values",

y = "Standardized conditional residuals"

) +

theme_classic(base_size = 12)

p_qq <- ggplot(diag_df, aes(sample = std_resid)) +

stat_qq(size = 1.5, alpha = 0.65) +

stat_qq_line(linewidth = 0.7) +

labs(

x = "Theoretical quantiles",

y = "Standardized conditional residuals"

) +

theme_classic(base_size = 12)

print(p_resid)

print(p_qq)

# **Reference**

- Donohue, M. C., Edland, S. D., & Hu, N. (2024). longpower: Sample Size Calculations for Longitudinal Data (Version 1.0.27) [Computer software]. Comprehensive R Archive Network (CRAN). <https://doi.org/10.32614/CRAN.package.longpower>.
- Goldberg, S. B., Bolt, D. M., & Davidson, R. J. (2021). Data Missing Not at Random in Mobile Health Research: Assessment of the Problem and a Case for Sensitivity Analyses. Journal of medical Internet research, 23(6), e26749. https://doi.org/10.2196/26749
- Matsumoto, K., Hamatani, S., Shiga, K., Mizuno, Y., Yoshinaga, N., Nakagawa, A., Kunisato, Y., Watanabe, K., Nagata, S., Maeno, A., Yokoo, M., Kimura, Y., Sunami, N., Ikeda, M., Kasai, M., Kamashita, R., & Nakamura, M. (2024). Effectiveness of unguided internet-based cognitive behavioral therapy for subthreshold social anxiety disorder in adolescents and young adults: Multicenter randomized controlled trial. JMIR Pediatrics and Parenting, 7, e55786. <https://doi.org/10.2196/55786>.
- Revelle, W. (2026). psych: Procedures for Psychological, Psychometric, and Personality Research(Version 2.6.5) [Computer software]. Comprehensive R Archive Network (CRAN). <https://doi.org/10.32614/CRAN.package.psych>.
- Rosseel, Y. (2012). lavaan: An R package for structural equation modeling. Journal of Statistical Software, 48(2), 1–36. <https://doi.org/10.18637/jss.v048.i02>.
